# Seasonality drives temporal niche partitioning of pelagic prokaryotes

**DOI:** 10.1093/ismejo/wrag062

**Published:** 2026-04-09

**Authors:** Fuyan Li, Andrew Burger, John M Eppley, David M Karl, Edward F DeLong

**Affiliations:** Daniel K. Inouye Center for Microbial Oceanography: Research and Education, University of Hawaiʻi, Mānoa, Honolulu, HI 96822, United States; Daniel K. Inouye Center for Microbial Oceanography: Research and Education, University of Hawaiʻi, Mānoa, Honolulu, HI 96822, United States; Daniel K. Inouye Center for Microbial Oceanography: Research and Education, University of Hawaiʻi, Mānoa, Honolulu, HI 96822, United States; Daniel K. Inouye Center for Microbial Oceanography: Research and Education, University of Hawaiʻi, Mānoa, Honolulu, HI 96822, United States; Daniel K. Inouye Center for Microbial Oceanography: Research and Education, University of Hawaiʻi, Mānoa, Honolulu, HI 96822, United States

**Keywords:** pelagic *Bacteria* and *Archaea*, periodic amplicon sequence variants (pASVs), annual cycles, seasonal ecotypes, temporal niche partitioning, biodiversity

## Abstract

Prokaryote seasonality is well documented in lakes, coastal areas, and inland seas, yet microbial annual periodicity in diverse open ocean settings is less well characterized. Here, we report seasonality in pelagic prokaryotes from open ocean depth profiles collected over 8.5 years in the North Pacific Subtropical Gyre. At depths shallower than 150 m, we found significant annual cycling in >62% of all pelagic prokaryote taxa. Although the proportion of seasonally cycling taxa diminished at depths >150 m, annual periodicity to depths of 4000 m was observed in some taxa. Even among closely related prokaryotes in the same clade, variable seasonal maxima were observed, a sub-clade-level temporal niche partitioning referred to here as seasonal ecotypes. One prevailing trend in the euphotic zone was a seasonal shift from surface water abundance maxima in winter, to spring and summer annual peaks at greater depths. In the upper mixed layer, most *Prochlorococcus* seasonal ecotypes had winter abundance maxima, while bacterial heterotrophs typically peaked in summer. In the euphotic subsurface, common seasonal ecotypes included *Prochlorococcus* having spring or summer maxima, and heterotrophic and chemolithoautotrophic prokaryotes having winter or fall maxima. At meso- and bathypelagic depths, most prokaryotes exhibited winter, spring, or fall seasonal maxima, alongside annual peaks of surface-dwelling photoautotrophs exported to the deep sea. The results indicate that seasonal ecotype annual cycling is a central feature of open ocean prokaryote communities, and suggest that subtle yet predictable seasonal niche partitioning helps drive and sustain prokaryote genetic diversification throughout the open ocean’s interior.

## Introduction

The North Pacific Subtropic Gyre (NPSG) represents one of Earth’s largest biomes, characterized by its year-round stratified surface waters. These conditions isolate nutrient-poor sunlit upper water column from deeper waters that have elevated nutrient concentrations, but limited light penetration [1]. Although bacterial and archaeal communities comprise a large fraction of biomass and drive critical biogeochemical cycles in the open ocean, it is less well known how these communities may vary seasonally throughout the ocean water column. Time-series studies can illuminate details of natural history, annual succession, and temporally regulated biotic interactions and activities. Constraining these temporal dynamics may also help to inform models that aim to predict microbial community resilience as well as structural and functional variability, in the context of environmental change.

The seasonality of planktonic prokaryotes has been documented in several freshwater lakes, coastal regions, and inland seas [2–6]. For example, strong seasonality of microbial communities in a 20-year time-series of a small eutrophic temperate lake has recently been reported [7, 8]. Here, annual cycles were in part driven by winter lake freezing, spring ice-melt with increased daylight and mixing, summer water column stratification with seasonal temperature maxima, and decreasing temperature, day length, and increased mixing in fall [7, 8]. In the Arctic and Southern Oceans, wintertime periods of ice cover and darkness, followed by ice melt and high solar irradiance in late spring and summer, can trigger blooms of algae and bacteria in surface waters [9, 10]. Coastal marine studies have consistently shown seasonal oscillation of coastal and inland sea microbial communities [2], including in the San Pedro Channel [11], the western English Channel [12], and the Northwestern Mediterranean Sea [13].

Due to their remote location and associated logistical challenges, seasonality of microbial populations in open ocean ecosystems is less well documented. Longstanding open ocean time series efforts such as the Hawai‘i Ocean Time-series program at Station ALOHA (22°45′N, 158°W) [1], and the Bermuda Atlantic Time-series Study (BATS) [14], have been systematically addressing these challenges for over 35 years. Pioneering studies of microbial community seasonality at BATS for example, have demonstrated pronounced seasonality in that habitat [3, 14–16]. There, the annual periodicity of prokaryote communities is strongly driven by wintertime deep-water mixing, and includes annual spring blooms of some species, followed by stabilizing of community structure in well-stratified waters during summer and fall [15, 16]. In contrast, at Station ALOHA in the NPSG deep-water winter mixing is stochastic and not as strongly expressed as in other habitats, so seasonality is more cryptic among surface water prokaryotes [17, 18]. Although physical variability in the NPSG is less pronounced, annual periodicity of some prokaryotes there, including *Crocosphaera* and other nitrogen-fixing cyanobacteria, has been well documented [19, 20]. Blooms of cyanobacterial diazotrophs in the NPSG in summer help fuel annual cycles of export to the deep-sea, underscoring the importance of seasonal cycles in open ocean habitats [1, 21, 22]. Likewise, picoeukaryote assemblages in the NPSG also exhibit annual cycles in the euphotic zone, with their seasonality explaining ~25% of the eukaryote microbial community variability at Station ALOHA in the NPSG [23].

Despite prior work, multiple-year studies of the annual periodicity of pelagic prokaryotes in perennially stratified oligotrophic habitats like the NPSG are rare. To address this knowledge gap, in this study we sought to address the following questions: (i) How prevalent and predictable is annual periodicity among surface water prokaryotes in the NPSG?; (ii) Is annual periodicity restricted to only specific taxa or physiological types?; (iii) How deep in the water column does prokaryote annual periodicity occur?; and (iv) Does prokaryote annual periodicity and surface primary production export in upper water column impact annual cycles of the deep-sea ecosystem? To address these questions, we conducted an 8.5-year time-series analysis of planktonic prokaryotes from surface waters to 4000 m. Prokaryotes were sampled approximately monthly at 20 depths (5–4000 m) from November 2014 to May 2023, resulting in an unprecedented 1367 discrete open ocean time-series DNA samples. Pelagic prokaryotes were characterized via 16S rRNA PCR amplification and gene sequencing, followed by periodicity analyses of the whole community, as well as individual amplicon sequence variants (ASVs). Periodic ASVs (pASVs) were detected using nonparametric detection of both symmetric and nonsymmetric rhythms in the RAIN (Rhythmicity Analysis Incorporating Non-parametric method) package [24] (*P* value ≤.05, see Methods), and those ASVs showing statistically significant annual periodicity are hereafter referred to as “pASVs”. Our data and analyses demonstrate that robust seasonal cycling is a common feature of pelagic prokaryotes throughout the NPSG water column in the NPSG. Furthermore, although prokaryote ecotypes (closely related phylogenetic variants having distinct spatial distributions with respect to depth or latitude that reflect unique niche differentiation) are well known, the existence of “temporal ecotypes” is less well documented. We show here that “seasonal ecotypes”, defined as closely related phylogenetic variants having annually recurring seasonal abundance maxima, are a common feature of many bacterial and archaeal lineages in the open ocean.

## Materials and methods

### Sampling

**Figure 1:**
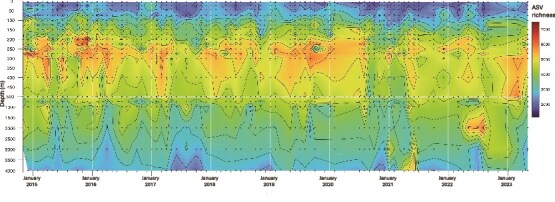
The total number (richness) of planktonic prokaryote ASVs at each depth sampled across the time series. The upper 500 m shows depth increments of 50 m, whereas the 500–4000 m depth range displays increments of 500 m. The contour interval is 500 ASVs for both the upper 500 m and the 500–4000 m depth ranges. Black dots represent individual sample points. White lines depict January of each year in the sample collection.

Water column suspended particle samples (including free-living cells) were collected approximately monthly from November 2014 through May 2023 on Hawai‘i Ocean Time-series (HOT) cruises at Station ALOHA (https://hahana.soest.hawaii.edu/hot/hot-dogs/crssum.html). The sampling of suspended water column particles during November 2014 through November 2016, and subsequent DNA extraction, 16S rRNA gene amplicon library construction, and sequencing, have been previously described [25]. A total of 2 l seawater from depths of 5–175 m and 4 l seawater from depths between 200 and 4000 m were collected by filtering through 0.2 μm Supor filters (Pall Life Sciences, Port Washington, NY, USA) without prefiltration. A total of 1367 suspended water column samples from 74 different time points collected over 8.5 years were included for downstream processing (Fig. 1).

### DNA extraction and amplicon sequencing

DNA extraction of water column samples was performed using Qiagen plant minikit and a QIAcube (Qiagen, Valencia, CA, USA). The “universal” primers 515F (5′-GTGYCAGCMGCCGCGGTAA-3′) and 806RB (5′-GGACTACNVGGGTWTCTAAT-3′) were used to amplify the V4 region of 16S rRNA gene, as described previously [25]. 5Prime HotMasterMix (Quantabio, Beverly, MA, USA) was used for amplification of 16S rRNA gene from samples collected during November 2014 through February 2020, and thereafter 5Prime HotMasterMix was discontinued from manufacturing. The rest of samples were conducted with Platinum Hot Start PCR Master Mix (Invitrogen by Thermo Fisher Scientific, Vilnius, LT, Lithuania). Amplicon purification was conducted with AMPure XP beads (Beckman Coulter, Brea, CA, USA). The purified amplicons were sequenced on a MiSeq System (Illumina). All 16S rRNA gene amplicon sequences have been deposited in the NCBI Sequence Read Archive under project number PRJNA352737 (Station ALOHA suspended prokaryote amplicons).

### Amplicon sequence processing

The 16S rRNA gene amplicon sequences were processed as previously described [25]. Low quality scores at the 12th and 21st base positions in reverse reads were observed in suspended water column samples during November 2015 to April 2016 and May 2017 to February 2019. The two nucleotide bases were therefore masked from all reverse reads. All reads were trimmed with Trimmomatic v0.36 [26], and split into individual sample files using QIIME [27]. Each sequencing run was processed with R package DADA2 [28] (1.14.1) using default settings, except for truncation length parameters (length of forward reads: 180, length of reverse reads: 100). For processing of each sequencing run using DADA2, samples were pooled together to maximize generating ASVs. Subsequently, ASVs identified from each sequencing run were merged into a single, non-redundant ASV table and chimeras were removed in DADA2 using default settings. Two ambiguous nucleotides (“N”) were placed at the 12th and 21st base positions from the end of ASV sequence. Taxonomy was assigned to each ASV sequence using assignTaxonomy() in DADA2 with the SILVA 16S rRNA gene reference database (v138.1) [29]. All *Prochlorococcus* ASVs were designated to *Prochlorococcus* MIT9313. *Prochlorococcus*, SAR11, and SAR202 ecotypes were further annotated in downstream analyses.

Based on non-metric multidimensional scaling compositions of suspended prokaryote ASVs and taxonomic profiles from the water column, 36 samples from 3 out of 74 cruises were found to be mislabeled and were corrected for the downstream analysis (Supplementary Table 1, Supplementary Fig. 1).

### *Prochlorococcus*, SAR11, and SAR202 ASV ecotype annotation

*Prochlorococcus* pASVs were assigned to *Prochlorococcus*_A, *Prochlorococcus*_B, *Prochlorococcus*_C, and *Prochlorococcus*_P (referred to *Prochloroccus* clades in GTDB database) using IdTaxa() [30] in DADA2 with the GTDB 16S rRNA reference database (r220, April 2024) [31]. *Prochlorococcus* pASVs were also annotated with the ProPortal-ASV-Annotation pipeline (https://github.com/jcmcnch/ProPortal-ASV-Annotation). Twenty one bases including two ambiguous nucleotides were removed from the end of each *Prochlorococcus* pASV sequence obtained from the above DADA2 assignTaxonomy() analysis. The trimmed sequences were assigned to HLI, HLII, LLI, LLII-III, and LLIV with 100% nucleotide identity and 100% coverage. To increase the accuracy of annotation, the ecotype assignment to each *Prochlorococcus* pASV was attained when two or more matches were identical. The depth-averaged profiles of the *Prochlorococcus* pASVs were clustered into four groups. *Prochlorococcus*_A pASVs were separated into two subgroups, *Prochlorococcus*_A_a (HLII) and *Prochlorococcus*_A_b (HLI). *Prochlorococcus*_B (LLI) pASVs formed a single group. *Prochlorococcus*_C and *Prochlorococcus*_P (LLII-III and LLIV) pASVs were grouped together and referred to as LLII-III-IV. The *Prochlorococcus* pASVs were therefore annotated to four ecotype groups, HLII, HLI, LLI, and LLII-III-IV.

SAR11 and SAR202 ASVs obtained from the above DADA2 assignTaxonomy() analysis were assigned to their ecotypes using PythonAssigner (v0.9, https://github.com/BIOS-SCOPE/PhyloAssigner_python_UCSB/tree/main) with full-length 16S rRNA gene database for SAR11 and SAR202 [32].

### *Prochlorococcus* and SAR11 COG0012 metagenomic operational taxonomic units

The COG0012 gene sequences were retrieved from metagenomic data collected monthly at Station ALOHA from November 2014 to November 2017 (PRJNA352737) [33]. Metagenomic reads were assembled into contigs as previously described [33]. Genes were predicted from the contigs using prodigal [34] and grouped into clusters with mmseqs2 [35] with a cutoff of 95% nucleotide identity. Cluster representative genes from COG0012 were identified with eggnog_mapper [36] and assigned taxonomy using lastal [37] homology to the GTDB database (r220, April 2024) [31].

The GTDB *Prochlorococus* species were mapped to HL and LL ecotypes using ITS region similarity [38]. The GTDB SAR11 species were assigned to SAR11 ecotypes by annotating GTDB SAR11 16S rRNA gene sequences (r226) using PythonAssigner as described above. *Prochlorococus* and SAR11 COG0012 mOTUs were designated with *Prochlorococus* HL and LL ecotypes and SAR11 subclades, respectively.

### Detection of annual periodicity

**Figure 2:**
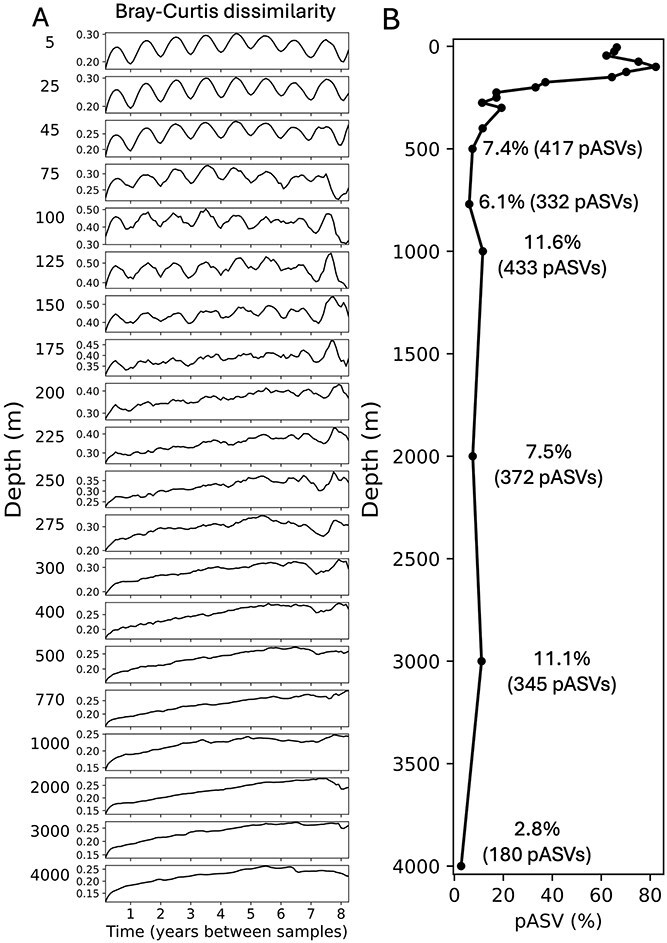
Annual periodicity of pelagic prokaryotes throughout the water column. (A) Mean value of Bray–Curtis dissimilarity of aggregate community ASV relative abundances between samples, as a function of the time lag between sample collections from 5 through 4000 m (the entire Bray–Curtis dissimilarity dataset is shown in Supplementary Fig. 3A). (B) The time-averaged percentage of pASVs relative to all ASVs at each depth sampled. At and below depths of 500 m percentages are displayed at selected depths, with total pASV numbers shown in parentheses (percentages and total numbers of pASVs in upper 400 m are shown in Supplementary Fig. 3D).

**Figure 3:**
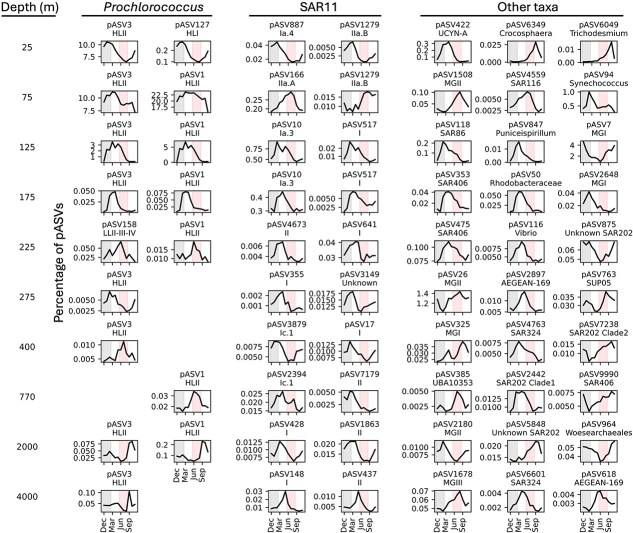
Monthly-averaged percentages of representative pASVs relative to the total ASVs for specific sampled depth. Percentages of pASVs are shown for *Prochlorococcus*, SAR11 and other dominant pelagic prokaryotes. Each individual curve displays statistically significant results (*P* value ≤.05, see Methods) from RAIN nonparametric analyses of rhythmicity. Gray highlights indicate wintertime periods (December, January, February), whereas red highlights display summertime periods (June, July, August).

The relative abundance of individual ASVs was used for periodicity analysis. Missing values were interpolated based on linear model with griddata() in MATLAB (R2024a). Pairwise Bray–Curtis dissimilarity between samples was detected for each depth, and their values were grouped based on time lag (Fig. 2A). For each depth, the mean value of each time lag group was calculated, and all of mean values as a function of time lag were tested for annual periodicity at the community level using implemented rain() package [24] in R (3.6.3) with the following settings: deltat = 1, period = 12, nr.series = 1, peak.border = c(0,1), verbose = FALSE, method = “independent”. RAIN analyses were performed for nonparametric rank test (summation of Mann–Whitney-U tests) of both symmetric and nonsymmetric rhythms, using Benjamini–Hochberg adjusted *P* values. Periodicity of individual ASV abundances was determined as a function of sample collection time (year and month) by screening for annual periodicity using the rain() R software package [24]. Annual periodicity was identified using a cutoff *P* value ≤0.05. Month-averaged percentages of representatives of pASVs were shown as a function of 12 months from January through December (Fig. 3).

**Figure 4:**
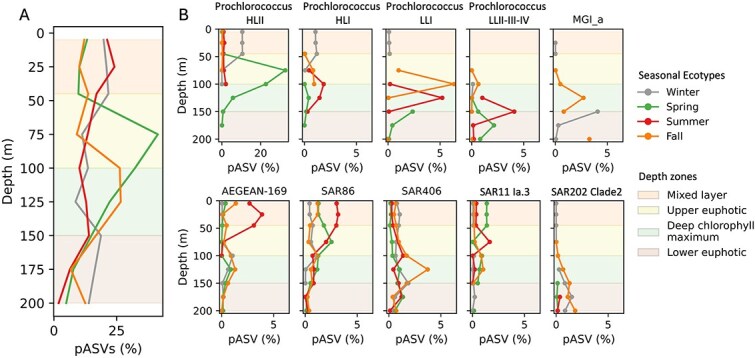
Depth profiles of time-averaged percentages of pASVs in the euphotic zone (~200 m). Shown are aggregate percentages of pASVs that peak in either winter (winter-peaking seasonal ecotype), spring (spring-peaking seasonal ecotype), summer (summer-peaking seasonal ecotype), or fall (fall-peaking seasonal ecotype) (A), and individual phylogenetic groups or ecotypes having seasonal peaks at different depths (B). Additional seasonally oscillating prokaryotic groups are shown in Supplementary Fig. 16. MGI_a: *Nitrosopelagicus*.

**Figure 5:**
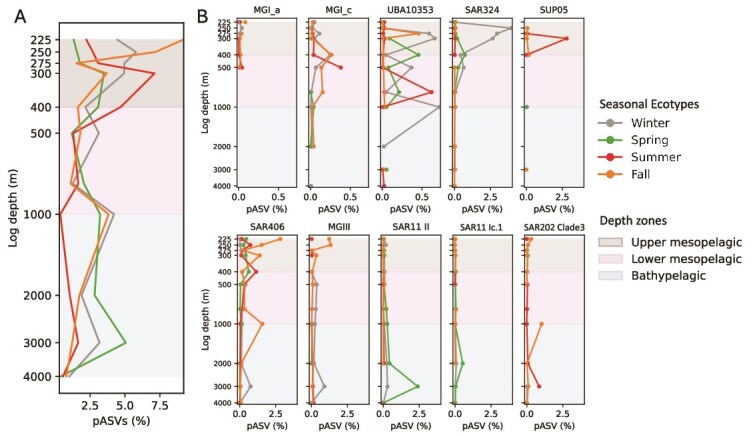
Depth profiles of time-averaged percentages of pASVs in the dark ocean (~200 m). Shown are aggregate percentages of pASVs that peak in winter (winter-peaking seasonal ecotype), spring (spring-peaking seasonal ecotype), summer (summer-peaking seasonal ecotype), or fall (fall-peaking seasonal ecotype) (A), and individual phylogenetic groups or ecotypes having seasonal peaks at different depths (B). Additional representatives of seasonally oscillating prokaryotic groups are shown in Supplementary Fig. 16. MGI_a: *Nitrosopelagicus*; MGI_c: unknown *Nitrosopumilaceae*.

All other analyses concerning seasonal peaks of pASVs, and their depth profiles (Figs 2B, 4, and  5) were conducted using measured values of pASVs instead of interpolated values. The number of ASVs was reported (Figs 1 and 2B), and all percentage values were the abundances of ASVs relative to the total abundance (Figs 2B, 3, 4, and  5). December, January, and February were wintertime; March, April, and May were springtime; June, July, and August were summertime; September, October, and November were fall (as defined at https://hahana.soest.hawaii.edu/hot/hot-dogs/documentation/prrseries.html). The seasonal peak was detected using season-averaged percentages of pASVs. The pASVs that peaked in winter were referred to winter-peaking seasonal ecotypes. Spring-, summer-, and fall-peaking seasonal ecotypes were identified here using the same approach. The total abundances of winter-, spring-, summer-, or fall-peaking seasonal ecotypes relative to the abundance of the total ASVs for each depth were calculated (Figs 4A, 5A). Depth profiles of winter-, spring-, summer-, and fall-peaking seasonal ecotypes were determined in dominant suspended prokaryotes (Figs 4B, 5B).

Similar interpolation and periodic analyses were applied to universal single-copy protein genes (COG0012) from *Prochlorococcus* and SAR11, using abundances of these metagenomic operational taxonomic units (mOTUS). Seasonal peaks were detected using season-averaged abundances of the universal single-copy protein gene (COG0012) mOTUs of selected taxa (*Prochlorococcus* and SAR11). Seasonally averaged gene abundances of individual seasonal ecotypes in these taxa relative to the total seasonal gene abundances from each taxon were then calculated.

## Results

### Pelagic prokaryotes exhibit robust seasonal cycles in the euphotic zone of the North Pacific Subtropic Gyre

Across all sample depths and time points, a total of 105 274 prokaryote 16S rRNA ASVs were identified, ranging from 1162 per sample to 7423 per sample (Fig. 1). The depth of maximum richness of prokaryote ASVs was relatively constant over time, centered around a depth of ~275 m (Fig. 1). The Shannon index of prokaryote ASVs found throughout the water column showed similar results (Supplementary Fig. 2). Seasonal oscillations in either community richness or the Shannon index of ASVs were also observed in the euphotic zone (Fig. 1, Supplementary Table 2). Pairwise Bray–Curtis dissimilarity of community composition between samples was calculated from planktonic prokaryote ASVs from each depth sampled, and annual periodicities were statistically evaluated using RAIN [24] (Fig. 2A, Supplementary Fig. 3A). The mean Bray–Curtis dissimilarity over 8.5 years reflected robust patterns of annual periodicity in the upper water column (5–150 m) for the whole prokaryote community and for heterotroph-like prokaryotes (RAIN *P* value <.0001; Fig. 2A, Supplementary Fig. 3A and B), whereas *Prochlorococcus*-specific community showed strong patterns of annual periodicity from 5 through 225 m (RAIN *P* value <.0001; Supplementary Fig. 3C). Prokaryote community annual periodicity declined at 175 m (RAIN *P* value = .00015), and diminished further below depths of 200 m (RAIN *P* value >.05; Fig. 2A, Supplementary Fig. 3A). Time-averaged percentages of pASVs (RAIN *P* value ≤.05) displayed depth-dependent decreases, with higher values (62.1%–82.3%) above 150 m, and lower values (37.2%–2.8%) below 150 m (Fig. 2B, Supplementary Fig. 3D and E). The same pattern was observed with respect to the total number of pASVs, that ranged from 1036–1381 pASVs at ~150 m and from 180 to 843 pASVs at depths below 150 m (Fig. 2B, Supplementary Fig. 3D and F). Collectively, these results support the existence of strong seasonality in most prokaryote taxa found in the upper water column (≥150 m). These analyses demonstrate strong seasonality and robust interannual resilience of suspended microbial communities in the upper photic zone of the NPSG.

Annual relative abundance maxima of different prokaryote pASVs occurred across all seasons and varied by depth (Figs 2B, 3). In the upper water column winter-peaks accounted for 19.8%–20.9% of all ASVs at 5–25 m, and decreased to 13.9%–16.3% at depths of 175–200 m (Fig. 4A, Supplementary Figs 3E, 4A). Spring-, summer-, and fall-peaks exhibited the similar depth-decreased trend (Fig. 4A, Supplementary Figs 3E, 4A). These results indicate that depth-dependent decrease of annual periodicity is a common feature of prokaryote communities in the NPSG. The data further show that summer- and winter-peaking pASVs were most abundant in the mixed layer (5–45 m), whereas those pASVs having maxima in spring or fall were most abundant in subsurface (75–150 m) (Fig. 4A, Supplementary Figs 3E, 4A).

We compared our results to previously reported, well-known annual cycles of pelagic cyanobacterial nitrogen-fixers in the NPSG [19, 20]. Among all diazotrophic cyanobacteria, *Crocosphaera, Richelia*, and *Trichodesmium* in surface waters (0–100 m) tended to peak in summer and fall, whereas *Atelocyanobacterium* (UCYN-A)’s maxima were detected in winter and spring across the same depth horizon (Supplementary Fig. 5). Accordingly, *Atelocyanobacterium* (UCYN-A) pASV422 reached maximal abundance in early spring at 25 m, whereas *Crocosphaera* pASV6349 and *Trichodesmium* pASV6049 had annual maxima in late summer/early fall at the same depth (Fig. 3). These results are consistent with previously reported seasonal variations of cyanobacterial nitrogen-fixers that leveraged nifH gene qPCR analyses [19, 20].

### Seasonal cycles of prokaryote amplicon sequence variants occur at meso- and bathypelagic depths

Although prokaryote annual periodicity was attenuated below the photic zone (Fig. 2; Supplementary Fig. 3), seasonal cycles of specific taxa in the deep-sea were apparent even at the greatest depths sampled (4000 m; Figs 2B, 3). The time-averaged percentage of total pASVs showed depth-decreased pattern in mesopelagic and bathypelagic zones, ranging from 11.3% to 19.3% between 225 and 400 m, and 2.8%–11.6% between 500 and 4000 m (Fig. 2B, Supplementary Fig. 3E). Winter-peaking pASVs ranged from 2.2 to 5.8% of total ASVs with a mean of 4.5% in the upper mesopelagic zone (225–400 m), from 1.2 to 4.2% (a mean of 2.9%) in the lower mesopelagic zone (500–1000 m), and from 1.0%–3.2% (a mean of 2.0%) in the bathypelagic zone (2000–4000 m) (Fig. 5A, Supplementary Figs 3E, 4B). Spring-, summer-, and fall-peaking pASVs showed the similar depth-dependent decrease in the deep-sea (Fig. 5A, Supplementary Figs 3E, 4B). Annual periodicity in richness or Shannon index of meso- and bathypelagic ASVs was also detected at multiple depths throughout the water column (Supplementary Table 1). Our results further indicate that pelagic prokaryote pASVs in the NPSG have characteristic winter and fall maxima in the upper mesopelagic zone; winter, spring, and fall annual peaks in the lower mesopelagic zone; and mostly winter and spring peaks in bathypelagic zone (Fig. 5A, Supplementary Figs 3E, 4B). Summer-peaking pASVs appeared less abundant than others in the lower mesopelagic and bathypelagic.

### Closely related periodic amplicon sequence variants having different seasonal maxima: seasonal ecotypes

In addition to well-known ecotypes having defined spatial distributions that reflect their depth-specific or latitude-variable physicochemical niches in space [18, 39, 40], we also observed seasonal oscillations among closely related ASVs in the same phylogenetic clade that varied in time (Figs 4B, 5B). For example, individual *Prochlorococcus* HLII ASV variants had different seasonal maxima that occurred across all four seasons in surface waters (Fig. 4B, Supplementary Figs 6, 7). Similarly, *Prochlorococcus* HLI was comprised of different genetic variants, which had maxima in different seasons in the upper euphotic zone (75–100 m) (Fig. 4B, Supplementary Figs 6, 7). Similar phenomena were observed among SAR11, SAR202, and other bacterial and archaeal phylogenetic clades (Figs 4B, 5B, Supplementary Figs 8, 9, 10, 11). Closely related strains in the same phylogenetic clade having annually recurring seasonal abundance maxima are referred to here to as seasonal ecotypes. We found that seasonal ecotypes were a common feature of many different pelagic bacterial and archaeal lineages throughout the NPSG water column (Figs 4B, 5B, Supplementary Figs 7, 9, 11).

Spatiotemporal patterns of pelagic prokaryote seasonal ecotypes were diverse and complex. Within the euphotic zone for example, *Prochlorococcus* HLII ASV1 relative abundance was aperiodic in the mixed layer, but had a spring maximum in the subsurface (Fig. 3, Supplementary Fig. 7C). *Prochlorococcus* HLII ASV3 in contrast peaked in winter in the mixed layer, and shifted to spring-peaking ecotypes in the euphotic zone subsurface water (Fig. 3, Supplementary Fig. 7C). Some heterotrophic bacteria like SAR11 Ia.3 pASV10 and SAR11 I pASV517, peaked in winter in the deep chlorophyl maximum, and shifted to a spring maximum in the lower euphotic zone (175–200 m) (Fig. 3). Another pattern that emerged included annually recurring export events. Specifically, *Prochlorococcus* HLII ASV1 and ASV3 had annual maxima in summer and fall at meso- and bathypelagic depths (Fig. 3, Supplementary Fig. 7C), presumably reflecting seasonal patterns of sinking particle export from the euphotic zone (see further discussion below).

**Figure 6:**
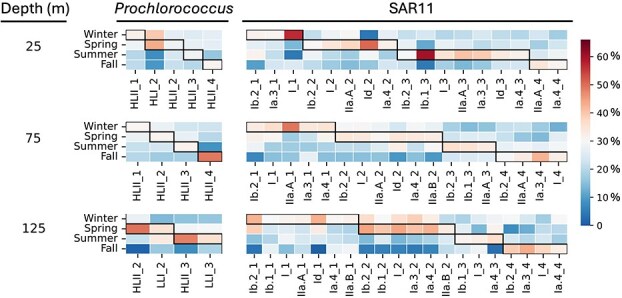
Heatmap of seasonally averaged percentages of annually periodic taxa inferred from universal single-copy protein sequence abundances (COG0012) for subclades of *Prochlorococcus* and SAR11. Shown on the x-axis are winter (_1), spring (_2), summer (_3), or fall (_4) peaking seasonal ecotypes of dominant *Prochlorococcus* and SAR11 subclades in the euphotic zone. The black rectangle represents the seasonal maxima for individual seasonal ecotypes.

Any method has potential to introduce its own inherent biases and caveats, and analysis of rRNA gene amplicon variants is no exception. Therefore, we employed a different analytical approach utilizing published metagenomes (as opposed to PCR amplicons) to further validate our findings using an independent method. Specifically, we conducted analyses based on single-copy ribosomal protein sequences (COG0012) using a “metagenomic OTU” (mOTU) approach [41], to confirm and extend ASV-based observations of seasonal ecotypes reported here. We recovered COG0012 protein coding-genes affiliated within *Prochlorococcus* and SAR11 from metagenome data previously collected at Station ALOHA (November 2014 through November 2017) [33]. As with the ASV data, seasonal ecotypes (RAIN *P* value ≤.05) were identified in both *Prochlorococcus* and SAR11 subclade variants of COG0012 (Fig. 6, Supplementary Fig. 12). For example, each of *Prochlorococcus* HLII, HLI, and LLI clades had unique seasonal ecotypes peaking in different seasons in the euphotic zone (Fig. 6A, Supplementary Fig. 12A). Similar patterns were observed for SAR11 I, Ia.3, Ia.4, IIa.A, and IIa.B subclades (Fig. 6B, Supplementary Fig. 12B). These results, independent of any PCR- or ASV-associated errors or biases, further confirm the existence of pelagic prokaryote seasonal ecotypes.

### Seasonal ecotypes exhibit temporal niche partitioning in the euphotic zone

In the euphotic zone, seasonal ecotypes of well-known photoautotrophic, chemolithoautotrophic, and heterotrophic bacterial and archaeal clades were common (Fig. 4B, Supplementary Figs 4C, 13). As expected, *Prochlorococcus* depth-variable seasonal ecotypes from HLII, HLI, LLI, and LLII-III-IV clades were all detected in the euphotic zone (Fig. 4B, Supplementary Figs 4C, 7A). Time-averaged percentages of *Prochlorococcus* HLII winter-peaking seasonal ecotypes comprised 10.5% of total ASVs in the mixed layer, along with less abundant HLI and LLI winter-peaking seasonal variants (1.1% and 0.1%, respectively; Fig. 4B, Supplementary Figs 4C, 7A). Lower in the water column, seasonal patterns changed, with *Prochlorococcus* seasonal ecotypes shifting towards spring-, summer-, and fall annual abundance maxima in the upper euphotic zone, deep chlorophyl maximum, and lower euphotic zone (Fig. 4B, Supplementary Figs 4C, 7A).

Chemolithoautotrophic ammonia-oxidizing Archaea were not abundant in NPSG surface waters [25, 42] (Supplementary Figs 14, 15), in part a result of photoinhibition of nitrifiers in this blue-water region [43]. *Nitrosopelagicus* (MGI_a) was the most abundant ammonia-oxidizing archaeal genus in the subsurface water of the euphotic zone (Supplementary Figs 14, 15), and seasonal variants in these depth regions for this archaeal clade were also detected mainly in the fall and winter (Fig. 4B).

Distinct seasonal ecotypes of heterotrophic lineages were also prevalent in the euphotic zone (Fig. 4B, Supplementary Figs 4C, 16A, 16B). The heterotrophic seasonal ecotypes peaking in winter surface waters (5–45 m) represented ~8.6% of total surface water ASV abundance (Supplementary Fig. 16A), and included SAR406 (0.9%), SAR324 (0.8%), *Rhodobacteraceae* (0.7%), SAR116 (0.7%), SAR86 (0.5%), and archaeal MGII clade (0.5%) (Fig. 4B, Supplementary Figs 4C, 16B). Heterotrophic seasonal ecotypes peaking in spring (~9.4%), summer (~19.5%), and fall (~11.0%) (Supplementary Figs 4C, 16A), included SAR11, SAR116, SAR86, the AEGEAN-169 marine clade, SAR324, and *Rhodobacteraceae* (Fig. 4B, Supplementary Figs 4C, 9A, 16B). Spring-peaking seasonal ecotypes in SAR11 clades I, Ia.3, and IIa.A were the most abundant among other seasonal ecotypes, whereas SAR11 Ia.4 seasonal ecotype peaking in summer displayed higher abundances, and SAR11 clades II, IIa.B, and IV fall-peaking seasonal ecotypes were more abundant (Fig. 4B, Supplementary Fig. 9A). Collectively, heterotrophic seasonal ecotypes in NPSG surface waters showed less seasonality among the winter-peaking variants, and more elevated levels of seasonal periodicity in those peaking in the summer.

Deeper within the euphotic zone, seasonal ecotypes in SAR11, SAR86, SAR406, SAR116, and archaeal MGII and MGIII peaked in spring (~8.2% of the total ASVs) and summer (~6.6%), whereas SAR202, SAR406, SAR11, S25-593, UBA10353, SAR324, archaeal MGII, SAR116, *Actinomarina*, AEGEAN-169 marine group, SAR86, and *Rhodobacteraceae* had winter maxima (~12.8%) (Fig. 4B, Supplementary Figs 4C, 9A, 11A, 16A, 16B). Fall seasonal ecotypes (~13.3%) in the subsurface included SAR324, SAR11, SAR406, SAR202, SAR86, *Actinomarina*, and AEGEAN-169 marine group (Fig. 4B, Supplementary Figs 4C, 9A, 11A, 16A, 16B). Seasonal ecotypes peaking in spring, summer, and fall in SAR11 subclades Ia.3, Ia.4, II, IIa.A, and IIb were more abundant, compared to winter-peaking seasonal ecotypes (Fig. 4B, Supplementary Fig. 9A). Fall- and winter-peaking seasonal ecotypes in SAR202 1, 2, 3, 4, and 5 clades had highest relative abundance (Fig. 4B, Supplementary Fig. 11A). Together, winter and fall seasonal ecotypes showed the largest contribution to annual periodicity in the subsurface euphotic zone waters, compared to spring and summer seasonal ecotypes.

### Seasonal ecotypes and temporal niche partitioning in the dark ocean can reflect *in situ* deep-sea microbial seasonality or surface-derived export

Although the overall seasonality of community composition diminished below the euphotic zone (Fig. 2A), bacterial and archaeal seasonal ecotypes were also present deep in the ocean’s interior (Figs 2B, 3, Supplementary Figs 4C, 13). For example, sulfur-oxidizing chemolithoautotrophic SAR324, SUP05, and UBA10353, and ammonia-oxidizing Archaea (*Nitrosopelagicus* (MGI_a), *Nitrosopumilus* (MGI_b), unknown *Nitrosopumilaceae* (MGI_c)) clades all included seasonal ecotype variants in the dark ocean (Fig. 5B, Supplementary Figs 4C, 16C, 16D). Of these, the seasonal ecotypes peaking in winter (0.97% of the total ASVs) were most abundant, compared to spring (0.19%), summer (0.34%), and fall (0.15%) ecotypes (Fig. 5B, Supplementary Figs 4C, 16C). *Nitrosopelagicus* (MGI_a) and unknown *Nitrosopumilaceae* (MGI_c) Archaea and SUP05 summer-peaking seasonal ecotypes had spikes at 500 and 300 m, respectively (Fig. 5B, Supplementary Figs 4C, 16D).

Upper mesopelagic heterotrophic prokaryote fall-peaking seasonal ecotypes (~3.1% of the total ASVs) were characterized by archaeal MGII and MGIII, SAR202 clades 1 and 2, SAR406, and SAR86 (Fig. 5B, Supplementary Figs 4C, 11B, 16E, 16F). SAR202 clades 2 and 5, SAR11 subclades I, Ia.3, IIb, and N2, SAR406, SAR86, MGII archaea, and Sva0996 seasonal ecotypes peaked in winter (~2.1%), spring (~1.9%), and summer (~2.6%) (Fig. 5B, Supplementary Figs 4C, 9B, 11B, 16E, 16F).

Lower mesopelagic and bathypelagic seasonal ecotypes peaking in winter and spring represented ~2.1% and ~2.3% of the total ASVs, respectively, and included AEGEAN-169 marine group, archaeal MGIII, SAR11 subclades I, Ic.1, II, IIb, and N2, SAR406, SAR86, KI89A, and *Woesearchaeales* (Fig. 5B, Supplementary Figs 4C, 9B, 16E, 16F). Summer- (~0.9%) and fall (1.6%)-peaking seasonal ecotypes were dominated by MGII and MGIII archaea, SAR202 clades 2, 3, 4, and 5, SAR406, and *Woesearchaeales* (Fig. 5B, Supplementary Figs 4C, 11B, 16E, 16F). Collectively, the annual periodicity of fall-peaking seasonal ecotypes in heterotrophic prokaryotes was more robust in the upper mesopelagic zone, whereas annual periodicity was more evident in spring- and winter-peaking seasonal ecotypes in the lower mesopelagic and bathypelagic zone. The time-averaged depth distributions of most of these deep-sea pASVs confirmed they were indigenous to the mesopelagic zone (Supplementary Fig. 14), suggesting the existence of robust seasonal cycles of *in situ* deep-sea prokaryotes.

Fall-peaking seasonal ecotypes were most prevalent at 225–250 m (Fig. 5A), and included bacterial heterotrophs such as SAR406, SAR86, and SAR202, and archaeal clades MGII and MGIII (Supplementary Fig. 13). Summer-peaking seasonal ecotypes had seasonal maxima at 300 m (Fig. 5A), and were primarily comprised of SUP05, Sva0996, archaeal MGII, SAR86, and SAR202 clades (Supplementary Fig. 13).

Surface derived export was evident in regularly recurring deep-water maxima of abundant cyanobacteria originating from sunlit waters, that occurred at mesopelagic and bathypelagic depths. For example, the most abundant ASVs across the entire water column were represented by *Prochlorococcus* HLII pASV1 and pASV3 variants, each having seasonal maxima in the euphotic zone (Fig. 3, Supplementary Fig. 7C). *Prochlorococcus* HLII pASV1 and pASV3 originating from surface waters also exhibited regular annual periodicity at mesopelagic and bathypelagic depths (Fig. 3, Supplementary Fig. 7C). Just below the photic zone, pASV3 had a seasonal maximum at 275 m (Fig. 3, Supplementary Fig. 7C). At greater depths in the mesopelagic, *Prochlorococcus* pASV1 and pASV3 had seasonal maxima in summer at 770–1000 m and 400 m, respectively (Fig. 3, Supplementary Fig. 7C). Fall seasonal maxima of these abundant cyanobacteria were also observed at bathypelagic depths (2000–4000 m) (Fig. 3, Supplementary Fig. 7C). These observations suggest that export processes that transport abundant surface-dwelling prokaryotes to the deep-sea, also have regularly recurring annual cycles.

## Discussion

Previous studies of pelagic prokaryote ecotypes have primarily focused on larger-scale, persistent depth, and biogeographic trends related to salinity, temperature, nutrient, or oxygen gradients, which may also drive ecotype niche partitioning [39, 40, 44–48]. Recently, more transient and localized physicochemical gradients induced by mesoscale eddies have also been shown to drive fine-scale niche partitioning in *Prochlorococcus* populations in the NPSG [31, 38].

Here, leveraging the increased spatial, temporal, and sequencing resolution, robust seasonality was observed among diverse prokaryotes at the community level (richness or Shannon index and Bray–Curtis distance among all ASVs), as well as among individual pASVs in the NPSG euphotic zone (Figs 1, 2, Supplementary Fig. 2, Supplementary Table 2). Although annual oscillations dampened with increasing depth within communities and among individual taxa, seasonality was evident even at the greatest depths sampled (4000 m; Figs 2B, 3).

The term “ecotype” refers to closely related species variants that have distinct ecological distributions reflecting their specific adaptations to unique niches [39, 49–51]. *Prochlorococcus* ecotypes for example, have been partitioned into several high-light (HL) and low-light (LL) clades that dominate in different depth horizons and biogeographic provinces in the ocean [39, 40] (Supplementary Figs 6, 14). *Prochlorococcus* HL ecotypes HLII and HLI grown in the laboratory have different temperature optima that in part reflects HLII prevalence in warm tropical waters, and the elevated abundance of HLI ecotypes in cooler, high latitude waters [39, 40] (Supplementary Figs 6, 14). Among those *Prochlorococcus* LL ecotypes in the subsurface, LLI ecotypes appear to better tolerate high light intensities, and have higher abundances closer to the surface [39, 40] (Supplementary Figs 6, 14). Subclades of SAR11 and SAR202 have also been identified as ecotypes having specific niche distributions in the water column [51, 52] (Supplementary Figs 8, 10). SAR11 Ia has been further subdivided into cold-water (Ia.1) and warm-water (Ia.3) ecotypes with distinct latitudinal distributions [51, 53]. Fresh (IIIb) and brackish water (IIIa) variants of the broader SAR11 clade have also been identified [47]. In the NPSG, we found that the SAR11 I, Ia.3, and II ecotypes were most abundant representatives of these subclades (Supplementary Figs 8, 14), whereas SAR202 ecotypes were more prevalent in subsurface waters [52, 54] (Supplementary Figs 10, 14). In addition to these previously reported phenomena, we show here that prokaryote seasonal ecotypes (closely related species variants that occupy distinct temporal niches) are an abundant and ubiquitous feature of the NPSG water column. Distinct seasonal prokaryote ecotypes occupying different temporal niches were observed throughout the water column.

Seasonal cycling of *Prochlorococcus*, SAR11, and SAR202 (among the most abundant pelagic bacteria in open ocean ecosystems at HOT and BATS) have been previously reported [18, 32, 53]. At BATS for example, SAR11 Ia.3 increased in relative abundance during summer, whereas SAR11 phylogenetic variants Ia.4, Ib, II, IIa.A, IIa.B, and IIb expressed an opposite annual oscillation [53]. The bacterial SAR202 1 clade likewise had an increased abundance in early winter in the North Atlantic Ocean [32]. Another study of PCR amplified internally transcribed spacer (ITS) sequences from *Prochlorococcus* populations reflected robust seasonal cycles at BATS, even though their annual periodicity in the NPSG at Station ALOHA in comparison was highly muted [17, 18]. These differences are likely due to the pronounced physicochemical seasonal variation at BATS (driven in part by annual winter-water overturn [16]) that does not regularly occur at similar magnitudes in the NPSG at Station ALOHA. In contrast, wind and solar irradiation emerged as two central factors shaping microbial diversity in NPSG surface waters [17].

The pelagic prokaryote seasonal ecotypes reported here exhibited diverse spatiotemporal patterns. Some did not exhibit any statistically significant annual periodicity. Among seasonally oscillating taxa (e.g. pASVs), some exhibited annual maxima in just a single season, at a single depth. Other pASVs peaked in two or more different seasons, but at different depths. A prevailing trend in the upper water column for some taxa having multiple seasonal peaks at different depths was a seasonal shift from surface water abundance maxima in winter, which transitioned to annual maxima at greater depths in spring and summer. This may reflect an annual winter to spring downward shift of the preferred niche space for some surface-dwelling prokaryotes, potentially related to seasonal shifts in temperature, nitrogen availability, or high light inhibition. At meso- and bathypelagic depths, annual periodicity appeared to reflect either *in situ* microbial growth, or a surface-derived export pattern.

In surface waters, winter- and summer-peaking seasonal ecotypes were most abundant (Fig. 4A), with winter ecotypes dominated by *Prochlorococcus* HLII and HLI variants, and summer ecotypes dominated by heterotrophic prokaryotes (Fig. 4B, Supplementary Fig. 4C). The increased relative abundance of surface *Prochlorococcus* HLII in winter could result from decreased competition for macronutrients with sympatric eukaryotic phytoplankton, which typically bloom in the summer. In contrast, during summer in the NPSG, solar irradiation and N_2_ fixation rates are elevated, supplying higher organic carbon and nutrients to the surface ecosystem and fueling the growth of heterotrophs, potentially helping shape the annual periodicities reported here.

In the euphotic zone subsurface, *Prochlorococcus* seasonal ecotypes were most abundant in the spring and summer (Fig. 4B), tracking increased solar irradiance in spring and summer [55]. *Prochlorococcus* LLI showed a maximum in fall-peaking seasonal ecotype at 100 m (Fig. 4B), as the photon flux decreases and isolumes (0.415 mol quanta/m^2^/d) shoal in fall [55]. In contrast, chemolithoautotrophic and heterotrophic bacteria and archaea were more abundant as winter- and fall-peaking seasonal ecotypes in euphotic zone subsurface waters (Fig. 4B, Supplementary Figs 4C, 16A, 16B). It is possible that nutrients (e.g. nitrate and nitrite, which were highest in fall at 75–200 m) (https://hahana.soest.hawaii.edu/hot/hot-dogs/bseries.html) acquired by photosynthesizers in spring and summer in the subsurface waters, are more available to chemolithoautotrophs and heterotrophs, as isolumes shoal in the winter and fall. The ^15^N isotopic composition of sinking particulate nitrogen collected at 150 and 4000 m showed that winter new production was based on nitrate [21, 56]. Subsurface heterotrophic prokaryotes may grow by uptake of nitrate, nitrite, and ammonium and export via entrainment into sinking particles [25], contributing to the sinking particulate exported nitrogen. Evidence for nitrate and nitrite assimilation has been reported in connection with genomes or isolates of multiple pelagic bacterial heterotrophs including SAR202 [52], SAR406 [57], SAR324 [58], *Rhodobacteraceae* [59], SAR11 [60], as well as archaeal MGII [61]. These organisms were dominant in winter- and fall-peaking seasonal ecotypes in the subsurface water (Fig. 4B, Supplementary Figs 4C, 16B).

In the dark portion of the NPSG, chemolithoautotrophic and heterotrophic prokaryotes were more abundant in spring-, fall-, and winter-peaking seasonal ecotypes (Fig. 5B, Supplementary Figs 4C, 16C, 16D, 16E, 16F). Here, a large, annually recurrent summer export pulse (SEP) occurs with production of rapid sinking particles when particulate carbon and nitrogen deep-sea export fluxes exceed 150% of annual mean fluxes in late summer [21]. During non-SEP periods, slower sinking particles are generated in the euphotic zone and undergo more efficient degradation during their slower downward transport, supplying more energy and nutrients to heterotrophs in the dark ocean [21]. These recurrent annual export patterns likely fuel the higher abundances of spring-, fall-, and winter-peaking seasonal ecotypes observed in the dark ocean, compared to the summer-peaking seasonal ecotypes prevalent in surface waters.

Regularly recurring seasonal export of dominant surface water taxa (in particular, *Prochlorococcus*) to the deep sea also emerged as an important ecosystem process in the NPSG. The most abundant *Prochlorococcus* ASVs from surface waters exhibited annual maxima in summer and fall seasons in the deep sea (Fig. 3, Supplementary Fig. 7C). These picophytoplankton can be exported out of the euphotic zone via the biological pump [62] as well as the “migrant pump” [63], primarily via rapidly sinking phytodetrital macroaggregates or fecal pellets [64]. Fragmentation and degradation of particles as they sink [65] may release *Prochlorococcus* from rapidly sinking particles into the suspended pool at depth. These seasonal cycles of surface-dwelling phototrophs detectable in the deep sea most likely reflect large-scale annually recurring summer export processes in the NPSG [21]. These data do not directly address particle sinking speeds, however because the specific export mechanisms are not currently well enough constrained. In the future, gaining better understanding of export pathways and their attenuation rates throughout the water column will help further enable estimation of the sinking speed of these phototrophs.

How planktonic species diversity can be so vast and sustained in seemingly homogeneous environments like the open ocean has been a long-standing conundrum in aquatic ecology [57]. The large amount of genetic and genomic “microdiversity” that has been demonstrated in many pelagic prokaryote lineages, raises even further questions [39, 51, 52, 66–68]. The notion of ecotypes [39, 49–51], wherein closely related species variants can occupy distinct ecological niches that change dynamically in space and time, in part helps to explain the “paradox of the plankton” [69, 70]. Additionally, theoretical consideration of fluctuation dependent mechanisms of coexistence can predict temporal conditions whereby multispecies communities stably persist [71]. Our observations here, showing that temporal ecotypes commonly occur among diverse lineages of closely related pelagic prokaryotes, provide further insight into how species diversity can be maintained among sympatric planktonic prokaryote species.

Annual periodicity (seasonality) is a fundamental property of many ecosystems. Although the NPSG may exhibit a less robust seasonal signal (e.g. a smaller range of annual temperature, day length, or light intensity fluctuations) than temperate habitats, seasonal cycling clearly impacts its biological and biogeochemical properties and dynamics [1, 19–23]. Theoretically, sympatric species diversity can be sustained if shared resources are used at different times, thereby minimizing competition [71]. In some habitats and species, utilization of resources across time may be heterogeneous, leading to annual variations in community diversity linked to shifts in resource availability [72]. Our observation that both Shannon and richness indices oscillated annually at most depths in the NPSG appears consistent with this trend. The seasonal periodicity we observed in community diversity at most depths, as well as that of individual seasonal ecotypes, suggest that annual changes in resource availability at any given depth may indeed be linked to pelagic prokaryote annual cycles [72]. The ability of any given habitat to sustain diverse species and stable communities relies in part on annually recurrent events (seasonality), along with the consistency of event recurrence from year to year (predictability) [73]. In the NPSG, the maintenance of temporal ecotypes may rely more on the high level of predictability of annual variation in this habitat, as opposed to the overall strength or dynamic range of seasonal fluctuations.

Many questions remain regarding the specifics of environmental variation and biological trait differentiation that may sustain annual temporal niche partitioning of prokaryote seasonal ecotypes. For example, which genomic features or physiological traits in any given lineage underpin the temporal differentiation of closely related seasonal ecotypes? Does variable gene content and resultant unique functional properties primarily drive temporal ecotype differentiation? Alternatively, does differential gene or protein expression across seasons contribute as a major driver of temporal differentiation? Does prokaryote annual seasonality influence ecosystem function and biogeochemical cycling in marine plankton? How do seasonal ecotypes vary between different ocean biogeographic provinces or biomes? Answers to these and related questions will provide deeper insight into the ecology, evolution and niche partitioning of indigenous planktonic prokaryotes throughout the ocean water column.

## Supplementary Material

wrag062_Supplemental_Files

## Data Availability

All data generated or analysed during this study are included in this published article.

## References

[1] Karl DM, Church MJ. Microbial oceanography and the Hawaii Ocean time-series programme. *Nat Rev Microbiol* 2014;12:699–713. 10.1038/nrmicro3333 25157695

[2] Raes EJ, Myles S, MacNeil L et al. Seasonal patterns of microbial diversity across the world oceans. *Limnol Oceanogr Letters* 2024;9:512–23. 10.1002/lol2.10422

[3] Treusch AH, Vergin KL, Finlay LA et al. Seasonality and vertical structure of microbial communities in an ocean gyre. *ISME J* 2009;3:1148–63. 10.1038/ismej.2009.60 19494846

[4] Raes EJ, Tolman J, Desai D et al. Seasonal bacterial niche structures and chemolithoautotrophic ecotypes in a North Atlantic fjord. *Sci Rep* 2022;12:15335. 10.1038/s41598-022-19165-w 36097189 PMC9468339

[5] Martin-Platero AM, Cleary B, Kauffman K et al. High resolution time series reveals cohesive but short-lived communities in coastal plankton. *Nat Commun* 2018;9:266. 10.1038/s41467-017-02571-4 29348571 PMC5773528

[6] Lambert S, Tragin M, Lozano JC et al. Rhythmicity of coastal marine picoeukaryotes, bacteria and archaea despite irregular environmental perturbations. *ISME J* 2019;13:388–401. 10.1038/s41396-018-0281-z 30254323 PMC6331585

[7] Rohwer RR, Hale RJ, Vander Zanden MJ et al. Species invasions shift microbial phenology in a two-decade freshwater time series. *Proc Natl Acad Sci USA* 2023;120:e2211796120. 10.1073/pnas.2211796120 36881623 PMC10089161

[8] Rohwer RR, Kirkpatrick M, Garcia SL et al. Two decades of bacterial ecology and evolution in a freshwater lake. *Nat Microbiol* 2025;10:246–57. 10.1038/s41564-024-01888-3 39753668

[9] Arrigo KR, Perovich DK, Pickart RS et al. Massive phytoplankton blooms under Arctic Sea ice. *Science* 2012;336:1408–8. 10.1126/science.1215065 22678359

[10] Williams TJ, Long E, Evans F et al. A metaproteomic assessment of winter and summer bacterioplankton from Antarctic peninsula coastal surface waters. *ISME J* 2012;6:1883–900. 10.1038/ismej.2012.28 22534610 PMC3446797

[11] Cram JA, Chow CE, Sachdeva R et al. Seasonal and interannual variability of the marine bacterioplankton community throughout the water column over ten years. *ISME J* 2015;9:563–80. 10.1038/ismej.2014.153 25203836 PMC4331575

[12] Gilbert JA, Steele JA, Caporaso JG et al. Defining seasonal marine microbial community dynamics. *ISME J* 2012;6:298–308. 21850055 10.1038/ismej.2011.107 PMC3260500

[13] Ferrera I, Auladell A, Balague V et al. Seasonal and interannual variability of the free-living and particle-associated bacteria of a coastal microbiome. *Environ Microbiol Rep* 2024;16:e13299. 10.1111/1758-2229.13299 39081120 PMC11289420

[14] Steinberg DK, Carlson CA, Bates NR et al. Overview of the US JGOFS Bermuda Atlantic time-series study (BATS): a decade-scale look at ocean biology and biogeochemistry. *Deep-Sea Res Part II: Topical Studies in Oceanography* 2001;48:1405–47. 10.1016/S0967-0645(00)00148-X

[15] Morris RM, Vergin KL, Cho JC et al. Temporal and spatial response of bacterioplankton lineages to annual convective overturn at the Bermuda Atlantic time-series study site. *Limnol Oceanogr* 2005;50:1687–96. 10.4319/lo.2005.50.5.1687

[16] Giovannoni SJ, Vergin KL. Seasonality in ocean microbial communities. *Science* 2012;335:671–6. 10.1126/science.1198078 22323811

[17] Bryant JA, Aylward FO, Eppley JM et al. Wind and sunlight shape microbial diversity in surface waters of the North Pacific subtropical gyre. *ISME J* 2016;10:1308–22. 10.1038/ismej.2015.221 26645474 PMC5029195

[18] Malmstrom RR, Coe A, Kettler GC et al. Temporal dynamics of Prochlorococcus ecotypes in the Atlantic and Pacific oceans. *ISME J* 2010;4:1252–64. 10.1038/ismej.2010.60 20463762

[19] Church MJ, Mahaffey C, Letelier RM et al. Physical forcing of nitrogen fixation and diazotroph community structure in the North Pacific subtropical gyre. *Glob Biogeochem Cycles* 2009;23:GB2020. 10.1029/2008GB003418

[20] Turk-Kubo KA, Henke BA, Gradoville MR et al. Seasonal and spatial patterns in diazotroph community composition at station ALOHA. *Front Mar Sci* 2023;10:1130158:1–16. 10.3389/fmars.2023.1130158

[21] Karl DM, Church MJ, Dore JE et al. Predictable and efficient carbon sequestration in the North Pacific Ocean supported by symbiotic nitrogen fixation. *Proc Natl Acad Sci USA* 2012;109:1842–9. 10.1073/pnas.1120312109 22308450 PMC3277559

[22] Dore JE, Letelier RM, Church MJ et al. Summer phytoplankton blooms in the oligotrophic North Pacific subtropical gyre: historical perspective and recent observations. *Prog Oceanogr* 2008;76:2–38. 10.1016/j.pocean.2007.10.002

[23] Rii YM, Karl DM, Church MJ. Temporal and vertical variability in picophytoplankton primary productivity in the North Pacific subtropical gyre. *Mar Ecol Prog Ser* 2016;562:1–18. 10.3354/meps11954

[24] Thaben PF, Westermark PO. Detecting rhythms in time series with RAIN. *J Biol Rhythm* 2014;29:391–400. 10.1177/0748730414553029 PMC4266694 25326247

[25] Li F, Burger A, Eppley JM et al. Planktonic microbial signatures of sinking particle export in the open ocean's interior. *Nat Commun* 2023;14:7177. 10.1038/s41467-023-42909-9 37935690 PMC10630432

[26] Bolger AM, Lohse M, Usadel B. Trimmomatic: a flexible trimmer for Illumina sequence data. *Bioinformatics* 2014;30:2114–20. 10.1093/bioinformatics/btu170 24695404 PMC4103590

[27] Caporaso JG, Kuczynski J, Stombaugh J et al. QIIME allows analysis of high-throughput community sequencing data. *Nat Methods* 2010;7:335–6. 10.1038/nmeth.f.303 20383131 PMC3156573

[28] Callahan BJ, McMurdie PJ, Rosen MJ et al. DADA2: high-resolution sample inference from Illumina amplicon data. *Nat Methods* 2016;13:581–3. 10.1038/nmeth.3869 27214047 PMC4927377

[29] Quast C, Pruesse E, Yilmaz P et al. The SILVA ribosomal RNA gene database project: improved data processing and web-based tools. *Nucleic Acids Res* 2012;41:D590–6. 10.1093/nar/gks1219 23193283 PMC3531112

[30] Murali A, Bhargava A, Wright ES. IDTAXA: a novel approach for accurate taxonomic classification of microbiome sequences. *Microbiome* 2018;6:140. 10.1186/s40168-018-0521-5 30092815 PMC6085705

[31] Parks DH, Chuvochina M, Waite DW et al. A standardized bacterial taxonomy based on genome phylogeny substantially revises the tree of life. *Nat Biotechnol* 2018;36:996–1004. 10.1038/nbt.4229 30148503

[32] Bolaños LM, Choi CJ, Worden AZ et al. Seasonality of the microbial community composition in the North Atlantic. *Front Mar Sci* 2021;8:624164:1–16. 10.3389/fmars.2021.624164

[33] Leu AO, Eppley JM, Burger A et al. Diverse genomic traits differentiate sinking-particle-associated versus free-living microbes throughout the oligotrophic open ocean water column. *MBio* 2022;13:e01569–22. 10.1128/mbio.01569-22 35862780 PMC9426571

[34] Hyatt D, Chen GL, LoCascio PF et al. Prodigal: prokaryotic gene recognition and translation initiation site identification. *BMC bioinformatics* 2010;11:119. 10.1186/1471-2105-11-119 20211023 PMC2848648

[35] Steinegger M, Söding J. MMseqs2 enables sensitive protein sequence searching for the analysis of massive data sets. *Nat Biotechnol* 2017;35:1026–8. 10.1038/nbt.3988 29035372

[36] Cantalapiedra CP, Hernández-Plaza A, Letunic I et al. eggNOG-mapper v2: functional annotation, orthology assignments, and domain prediction at the metagenomic scale. *Mol Biol Evol* 2021;38:5825–9. 10.1093/molbev/msab293 34597405 PMC8662613

[37] Kiełbasa SM, Wan R, Sato K et al. Adaptive seeds tame genomic sequence comparison. *Genome Res* 2011;21:487–93. 10.1101/gr.113985.110 21209072 PMC3044862

[38] Sheyn U, Poff KE, Eppley JM et al. Mesoscale eddies shape Prochlorococcuscommunity structure and dynamics in the oligotrophic open ocean. *ISME J* 2025;19:wraf106:1–13. 10.1093/ismejo/wraf106 40415184 PMC12236433

[39] Biller SJ, Berube PM, Lindell D et al. Prochlorococcus: the structure and function of collective diversity. *Nat Rev Microbiol* 2015;13:13–27. 10.1038/nrmicro3378 25435307

[40] Johnson ZI, Zinser ER, Coe A et al. Niche partitioning among Prochlorococcus ecotypes along ocean-scale environmental gradients. *Science* 2006;311:1737–40. 10.1126/science.1118052 16556835

[41] Mende DR, Boeuf D, DeLong EF. Persistent Core populations shape the microbiome throughout the water column in the North Pacific subtropical gyre. *Front Microbiol* 2019;10:2273. 10.3389/fmicb.2019.02273 31632377 PMC6779783

[42] Li F, Leu A, Poff K et al. Planktonic archaeal ether lipid origins in surface waters of the North Pacific subtropical gyre. *Front Microbiol* 2021;12:610675. 10.3389/fmicb.2021.610675 34589060 PMC8473941

[43] Qin W, Amin SA, Martens-Habbena W et al. Marine ammonia-oxidizing archaeal isolates display obligate mixotrophy and wide ecotypic variation. *Proc Natl Acad Sci USA* 2014;111:12504–9. 10.1073/pnas.1324115111 25114236 PMC4151751

[44] Thompson AW, van den Engh G, Ahlgren NA et al. Dynamics of Prochlorococcus diversity and Photoacclimation during short-term shifts in water column stratification at station ALOHA. *Front Mar Sci* 2018;5:488. 10.3389/fmars.2018.00488

[45] van den Engh GJ, Doggett JK, Thompson AW et al. Dynamics of Prochlorococcus and Synechococcus at station ALOHA revealed through flow cytometry and high-resolution vertical sampling. *Front Mar Sci* 2017;4:359. 10.3389/fmars.2017.00359

[46] Ulloa O, Henríquez-Castillo C, Ramírez-Flandes S et al. The cyanobacterium Prochlorococcus has divergent light-harvesting antennae and may have evolved in a low-oxygen ocean. *Proc Natl Acad Sci USA* 2021;118:e2025638118. 10.1073/pnas.2025638118 33707213 PMC7980375

[47] Lanclos VC, Rasmussen AN, Kojima CY et al. Ecophysiology and genomics of the brackish water adapted SAR11 subclade IIIa. *ISME J* 2023;17:620–9. 10.1038/s41396-023-01376-2 36739346 PMC10030771

[48] Berube PM, Rasmussen A, Braakman R et al. Emergence of trait variability through the lens of nitrogen assimilation in Prochlorococcus. *elife* 2019;8:e41043. 10.7554/eLife.41043 30706847 PMC6370341

[49] Turesson G . The genotypical response of the plant species to the habitat. *Hereditas* 1922;3:211–350. 10.1111/j.1601-5223.1922.tb02734.x

[50] Cohan FM . Towards a conceptual and operational union of bacterial systematics, ecology, and evolution. *Philos Trans Roy Soc Lond B Biol Sci* 2006;361:1985–96. 10.1098/rstb.2006.1918 17062416 PMC1764936

[51] Giovannoni SJ . SAR11 bacteria: the Most abundant plankton in the oceans. *Annu Rev Mar Sci* 2017;9:231–55. 10.1146/annurev-marine-010814-015934 27687974

[52] Saw JHW, Nunoura T, Hirai M et al. Pangenomics analysis reveals diversification of enzyme families and niche specialization in globally abundant SAR202 bacteria. *MBio* 2020;11:e02975–19. 10.1128/mBio.02975-19 31911493 PMC6946804

[53] Bolanos LM, Tait K, Somerfield PJ et al. Influence of short and long term processes on SAR11 communities in open ocean and coastal systems. *ISME Commun* 2022;2:116. 10.1038/s43705-022-00198-1 37938786 PMC9723719

[54] Landry Z, Swan BK, Herndl GJ et al. SAR202 genomes from the Dark Ocean predict pathways for the oxidation of recalcitrant dissolved organic matter. *MBio* 2017;8:e00413–7. 10.1128/mBio.00413-17 28420738 PMC5395668

[55] Letelier RM, Karl DM, Abbott MR et al. Light driven seasonal patterns of chlorophyll and nitrate in the lower euphotic zone of the North Pacific subtropical gyre. *Limnol Oceanogr* 2004;49:508–19. 10.4319/lo.2004.49.2.0508

[56] Böttjer D, Dore JE, Karl DM et al. Temporal variability of nitrogen fixation and particulate nitrogen export at station ALOHA. *Limnol Oceanogr* 2017;62:200–16. 10.1002/lno.10386

[57] Hawley AK, Nobu MK, Wright JJ et al. Diverse Marinimicrobia bacteria may mediate coupled biogeochemical cycles along eco-thermodynamic gradients. *Nat Commun* 2017;8:1507. 10.1038/s41467-017-01376-9 29142241 PMC5688066

[58] Boeuf D, Eppley JM, Mende DR et al. Metapangenomics reveals depth-dependent shifts in metabolic potential for the ubiquitous marine bacterial SAR324 lineage. *Microbiome* 2021;9:172. 10.1186/s40168-021-01119-5 34389059 PMC8364033

[59] Liang KYH, Orata FD, Boucher YF et al. Roseobacters in a sea of poly- and Paraphyly: whole genome-based taxonomy of the family Rhodobacteraceae and the proposal for the Split of the "Roseobacter clade" into a novel family, Roseobacteraceae fam. Nov. *Front Microbiol* 2021;12:683109. 10.3389/fmicb.2021.683109 34248901 PMC8267831

[60] Tsementzi D, Wu J, Deutsch S et al. SAR11 bacteria linked to ocean anoxia and nitrogen loss. *Nature* 2016;536:179–83. 10.1038/nature19068 27487207 PMC4990128

[61] Rinke C, Rubino F, Messer LF et al. A phylogenomic and ecological analysis of the globally abundant marine group II archaea (Ca. Poseidoniales Ord. Nov.). *ISME J* 2019;13:663–75. 10.1038/s41396-018-0282-y 30323263 PMC6461757

[62] Richardson TL, Jackson GA. Small phytoplankton and carbon export from the surface ocean. *Science* 2007;315:838–40. 10.1126/science.1133471 17289995

[63] Boyd PW, Claustre H, Levy M et al. Multi-faceted particle pumps drive carbon sequestration in the ocean. *Nature* 2019;568:327–35. 10.1038/s41586-019-1098-2 30996317

[64] Richardson TL . Mechanisms and pathways of small-phytoplankton export from the surface ocean. *Annu Rev Mar Sci* 2019;11:57–74. 10.1146/annurev-marine-121916-063627 29996063

[65] Nathan Briggs GDO . Hervé Claustre major role of particle fragmentation in regulating biological sequestration of CO2 by the oceans. *Science* 2020;367:791–3. 10.1126/science.aay1790 32054763

[66] Fraser C, Alm EJ, Polz MF et al. The bacterial species challenge: making sense of genetic and ecological diversity. *Science* 2009;323:741–6. 10.1126/science.1159388 19197054

[67] Shapiro BJ, Friedman J, Cordero OX et al. Population genomics of early events in the ecological differentiation of bacteria. *Science* 2012;336:48–51. 10.1126/science.1218198 22491847 PMC3337212

[68] Polz MF, Alm EJ, Hanage WP. Horizontal gene transfer and the evolution of bacterial and archaeal population structure. *Trends Genet* 2013;29:170–5. 10.1016/j.tig.2012.12.006 23332119 PMC3760709

[69] Hutchinson GE . The paradox of the plankton. *Am Nat* 1961;95:137–45. 10.1086/282171

[70] MacArthur R . Species packing and competitive equilibrium for many species. *Theor Popul Biol* 1970;1:1–11. 10.1016/0040-5809(70)90039-0 5527624

[71] Chesson P . Mechanisms of maintenance of species diversity. *Annu Rev Ecol Evol Syst* 2000;31:343–66. 10.1146/annurev.ecolsys.31.1.343

[72] Shimadzu H, Dornelas M, Henderson PA et al. Diversity is maintained by seasonal variation in species abundance. *BMC Biol* 2013;11:98. 10.1186/1741-7007-11-98 24007204 PMC3856667

[73] Tonkin JD, Bogan MT, Bonada N et al. Seasonality and predictability shape temporal species diversity. *Ecology* 2017;98:1201–16. 10.1002/ecy.1761 28144975

