# Supplementary material for: Seasonality drives temporal niche partitioning of pelagic prokaryotes
Source: ISME J. 2026 Apr 9;20(1):wrag062. doi: 10.1093/ismejo/wrag062 (PMC13082231; doi:10.1093/ismejo/wrag062)
Supplement: wrag062_Supplemental_Files [file wrag062_supplemental_files.zip › SupplFigureTable_wrag062.pdf]

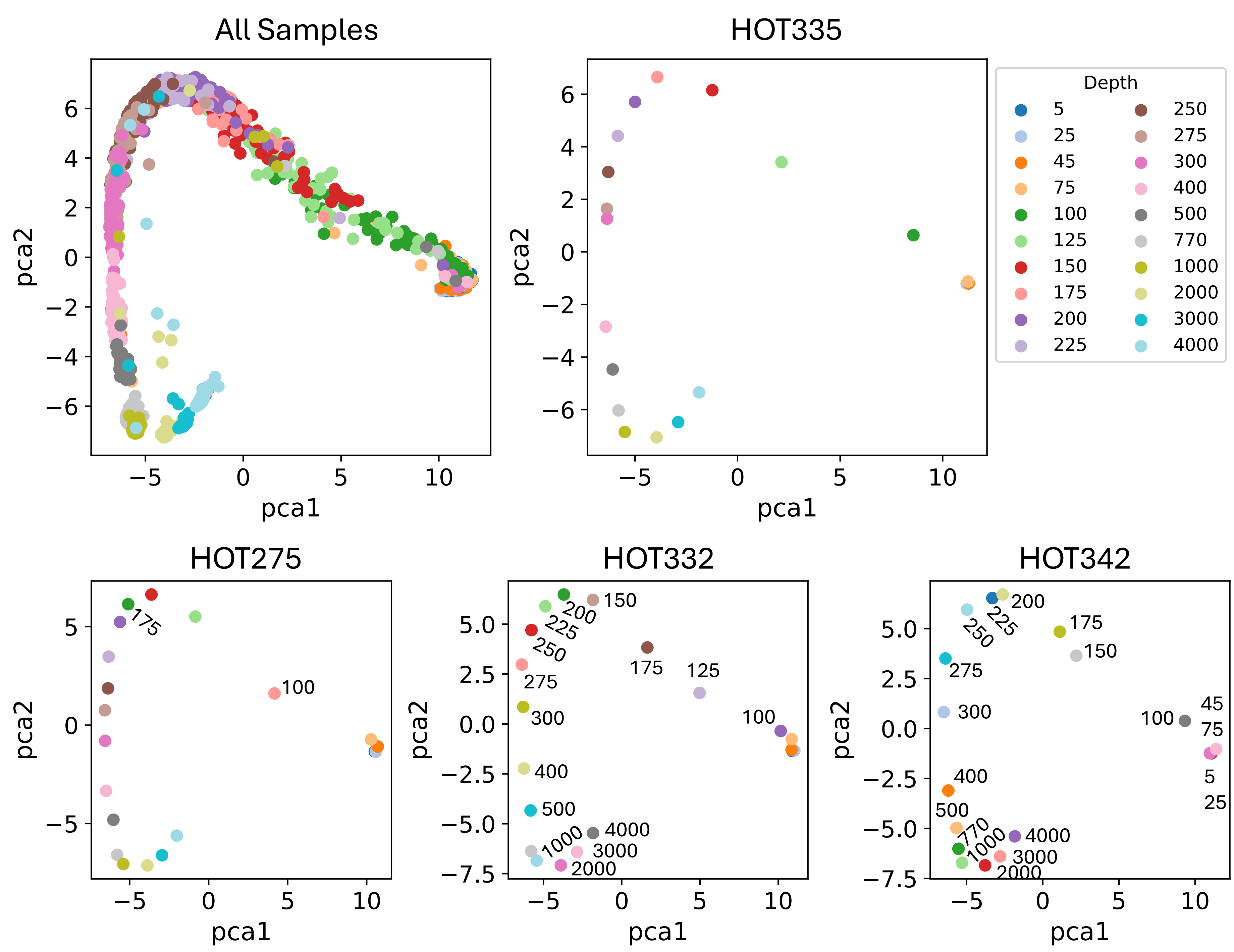

Supplementary Figure 1. NMDS analyses of all samples including mislabeled samples, one cruise (HOT335) having correct sample names, and three cruises (HOT275, HOT332, and HOT342) having mislabeled sample names. Depths were corrected and shown in each panel for HOT275, HOT332 and HOT342.

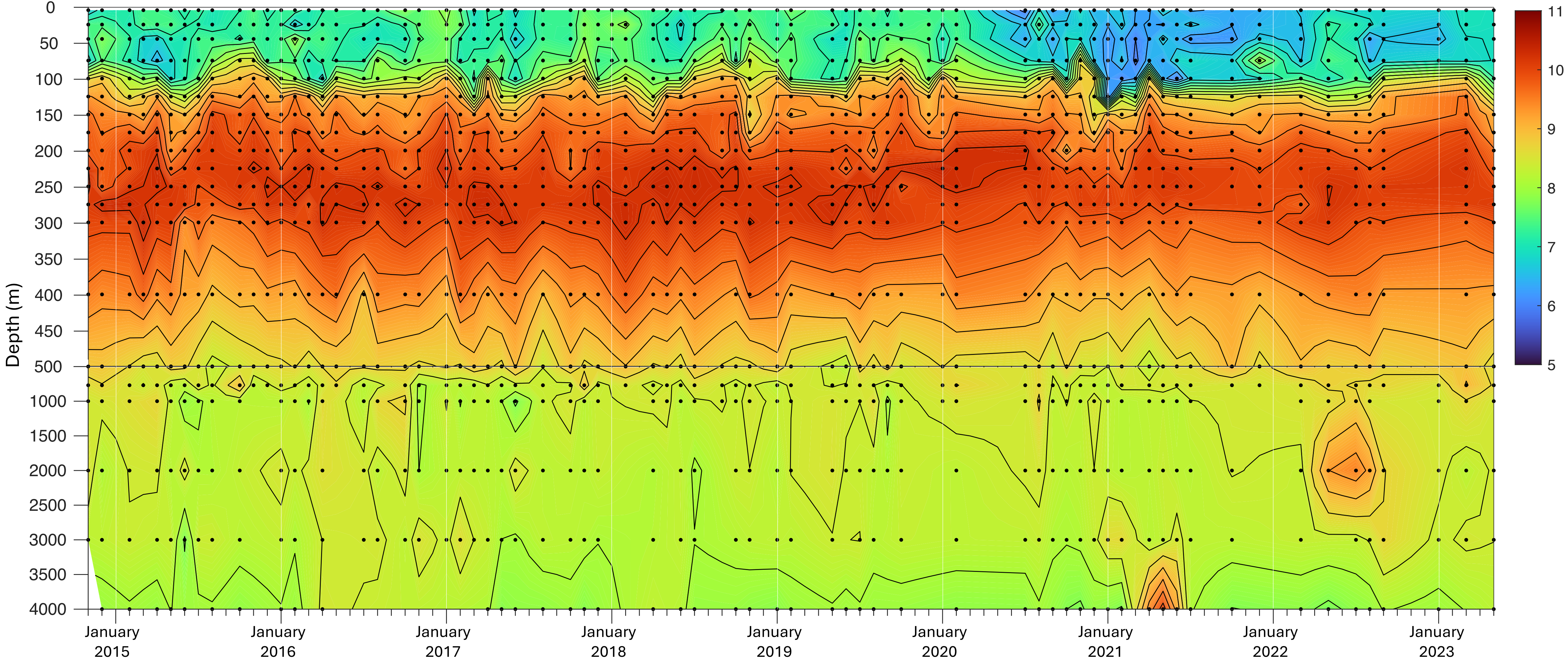

Supplementary Figure 2. Shannon index of planktonic prokaryote ASVs at each depth sampled across the time series. The upper 500 m has depth increments of 50 m, whereas the 500-4000 m depth range shows increments of 500 m. The contour interval is 0.3 for both the upper 500 m and the 500-4000 m depth ranges. Black dots represent sample points. White lines depict January of each year in the sample collection.

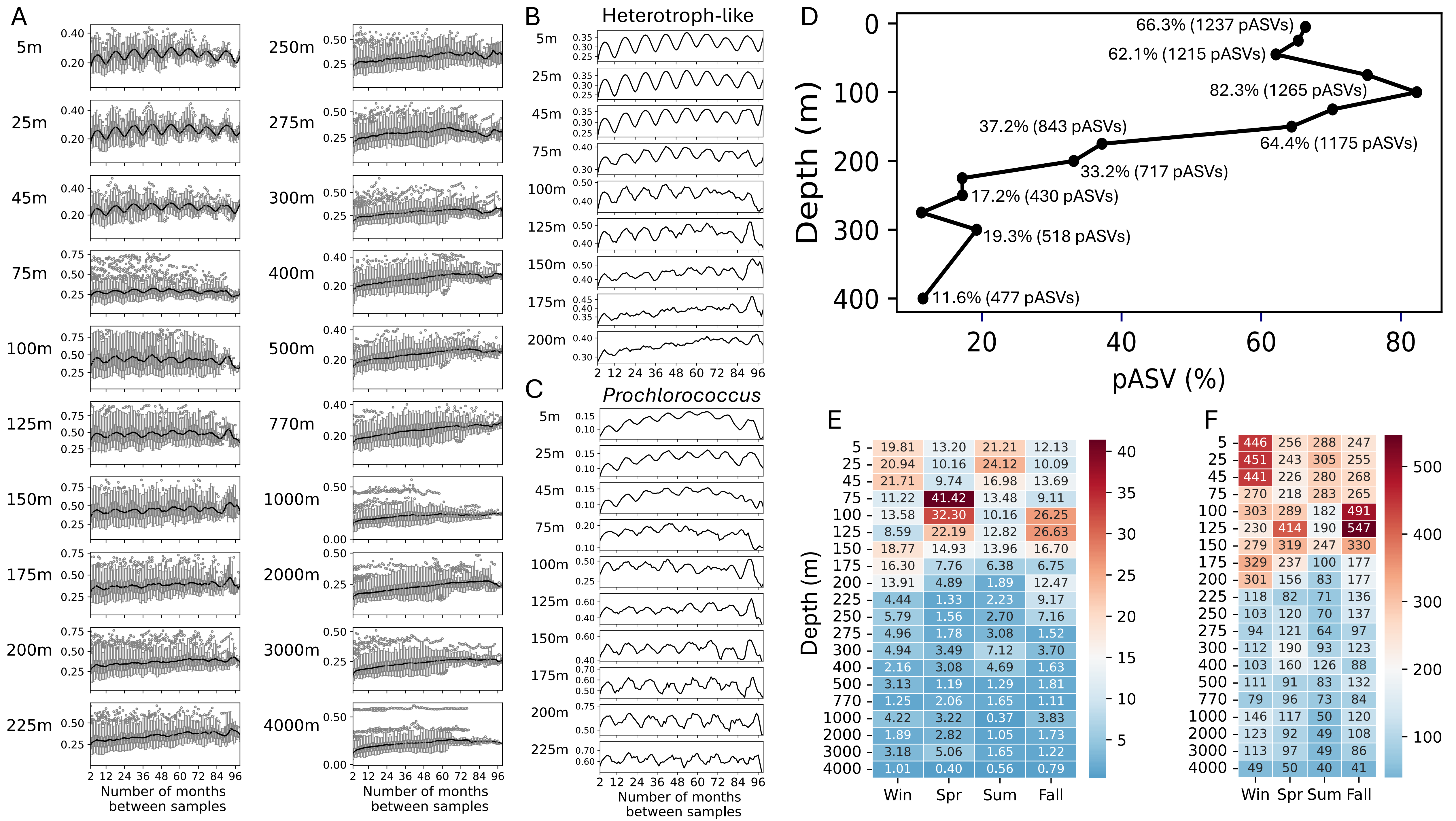

Supplementary Figure 3. Box plots for Bray-Curtis dissimilarity of ASV relative abundance between samples as a function of the time lag between sample collections from 5 through 4000 m for all prokaryote ASVs A), for heterotroph-like prokaryote ASVs B), and for *Prochlorococcus* ASVs C). 1.5IQR (Interquartile Range) was applied here. The grey circles are outliers. The black curve for each depth is mean value. D) the time-averaged abundance of the total pASVs relative to the total ASVs above 500 m. Percentages are indicated, along with total pASV numbers in parentheses. Heatmaps for depth profiles of winter-, spring-, summer-, and fall-peaking seasonal ecotypes in the pASV time-averaged abundance (%) relative to the total prokaryote community E) and in the total number of pASVs at each depth F).

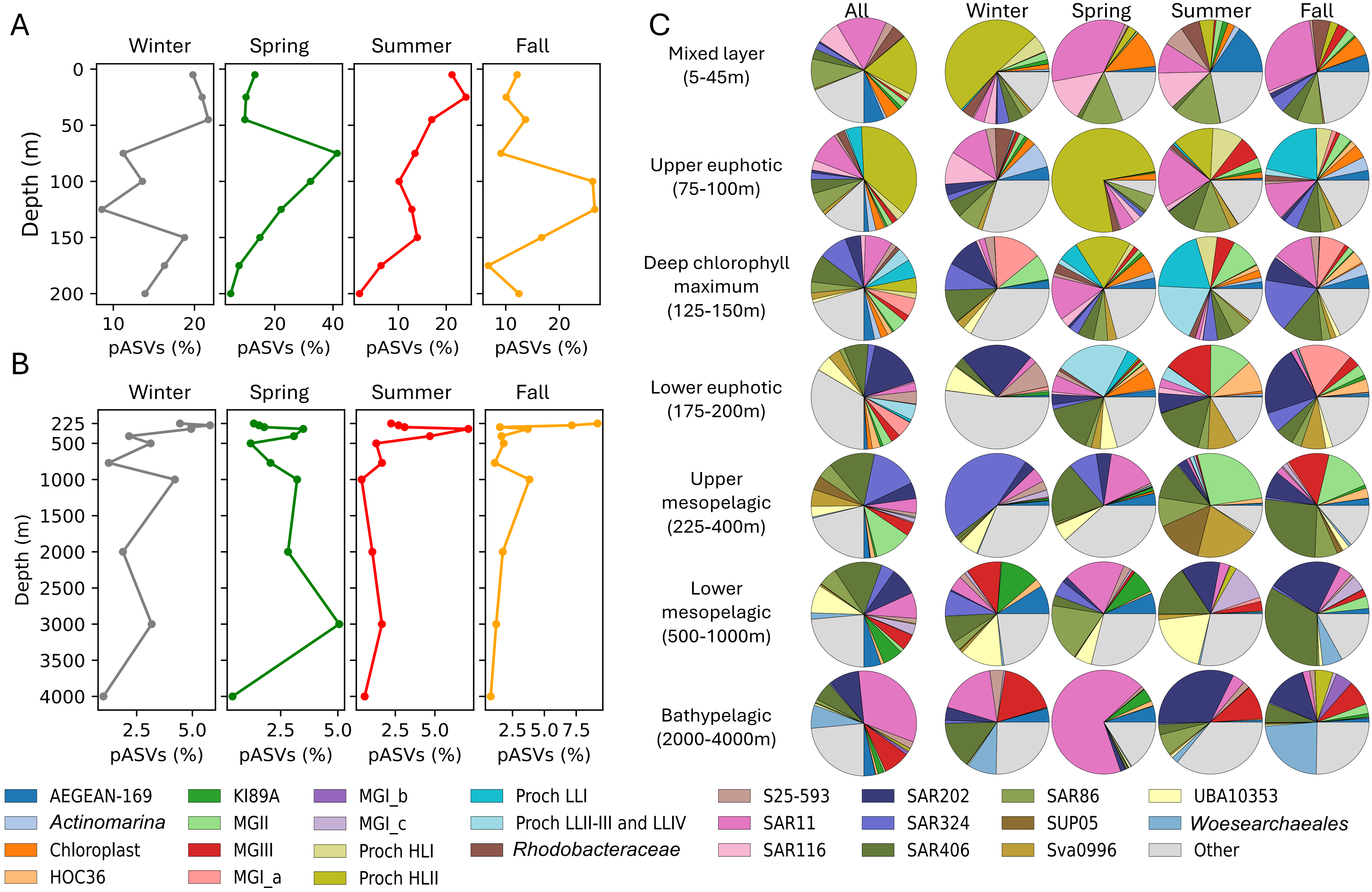

Supplementary Figure 4. Depth profiles of aggregate time-averaged relative abundances of seasonal ecotypes. Shown are pASVs that peak in winter (grey color, winter-peaking seasonal ecotype), spring (green color, spring-peaking seasonal ecotype), summer (red color, summer-peaking seasonal ecotype), and fall (orange color, fall-peaking seasonal ecotype), in the euphotic zone (~ 200 m) A) and dark ocean (200 m ~) B). C) The pASV taxonomic patterns of all, winter-, spring-, summer-, and fall-peaking seasonal ecotypes in dominant planktonic prokaryotes that show annual periodicity in different depth zones throughout the water column.

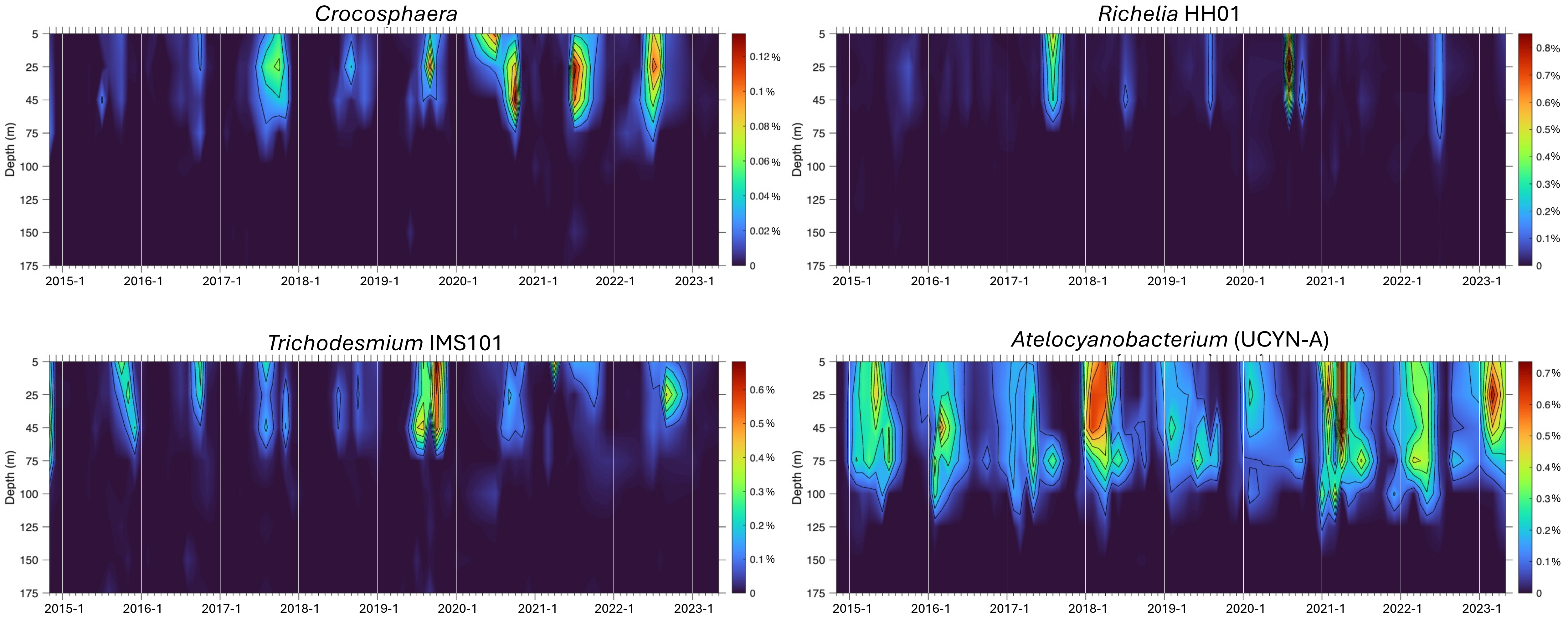

Supplementary Figure 5. Contour plots of ASV relative abundance of *Crocosphaera*, *Richelia*, *Trichodesmium* and *Atelocyanobacterium* (UCYN-A) in the euphotic zone. White lines depict January of each year in the sample collection.

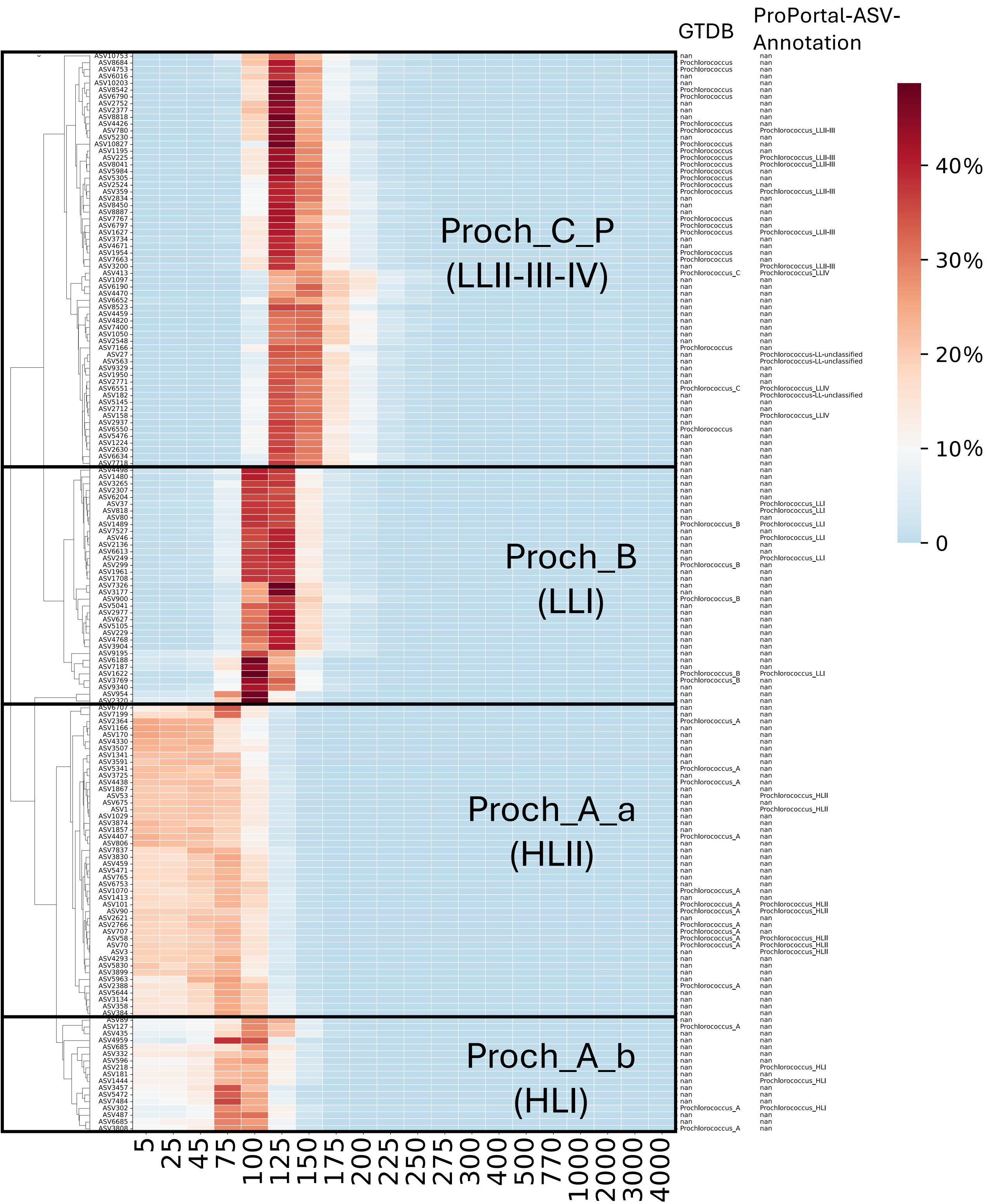

Supplementary Figure 6. Depth pattern of all *Prochlorococcus* pASVs relative to the total abundance of each pASV throughout the water column. The *Prochlorococcus* annotations were obtained using the GTDB database and ProPortal-ASV-Annotation (https://github.com/jcmcnch/ProPortal-ASV-Annotation).

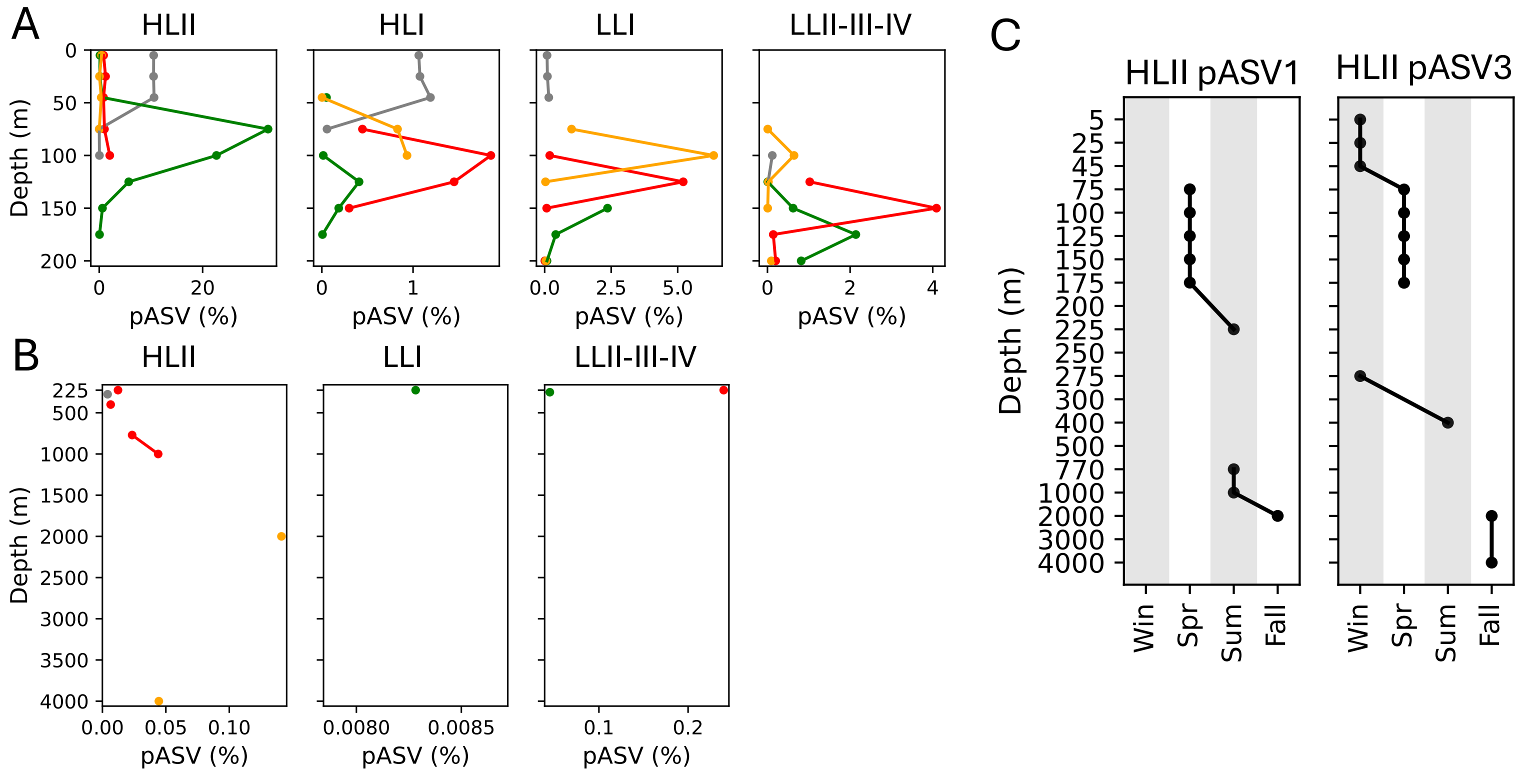

Supplementary Figure 7. Depth profiles of time-averaged percentages of *Prochlorococcus p*ASV ecotypes having winter (grey color), spring (green color), summer (red color) or fall (orange color) seasonal maxima in the euphotic zone A) or the dark ocean B). C) Depth shifts of seasonal ecotypes of *Prochlorococcus* HLII pASV1 and pASV3.

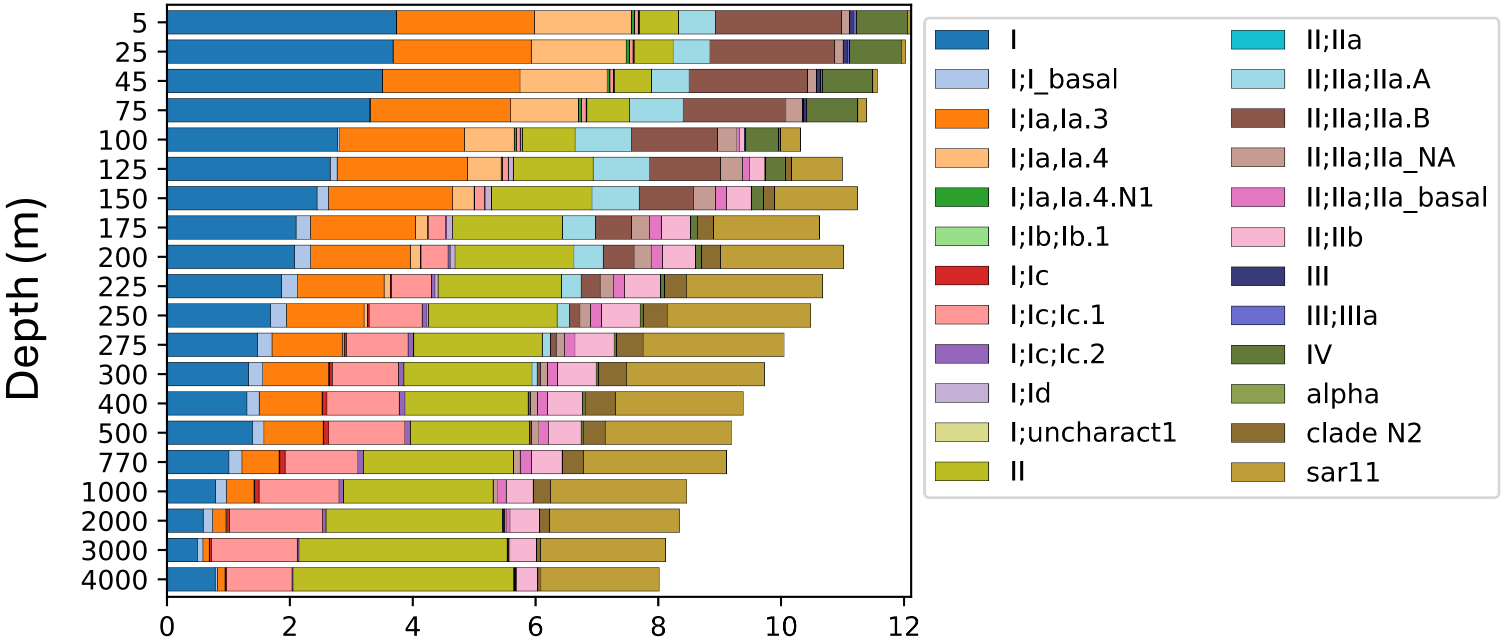

Supplementary Figure 8. Depth ASV patterns of SAR11 ecotypes detected in the water column.

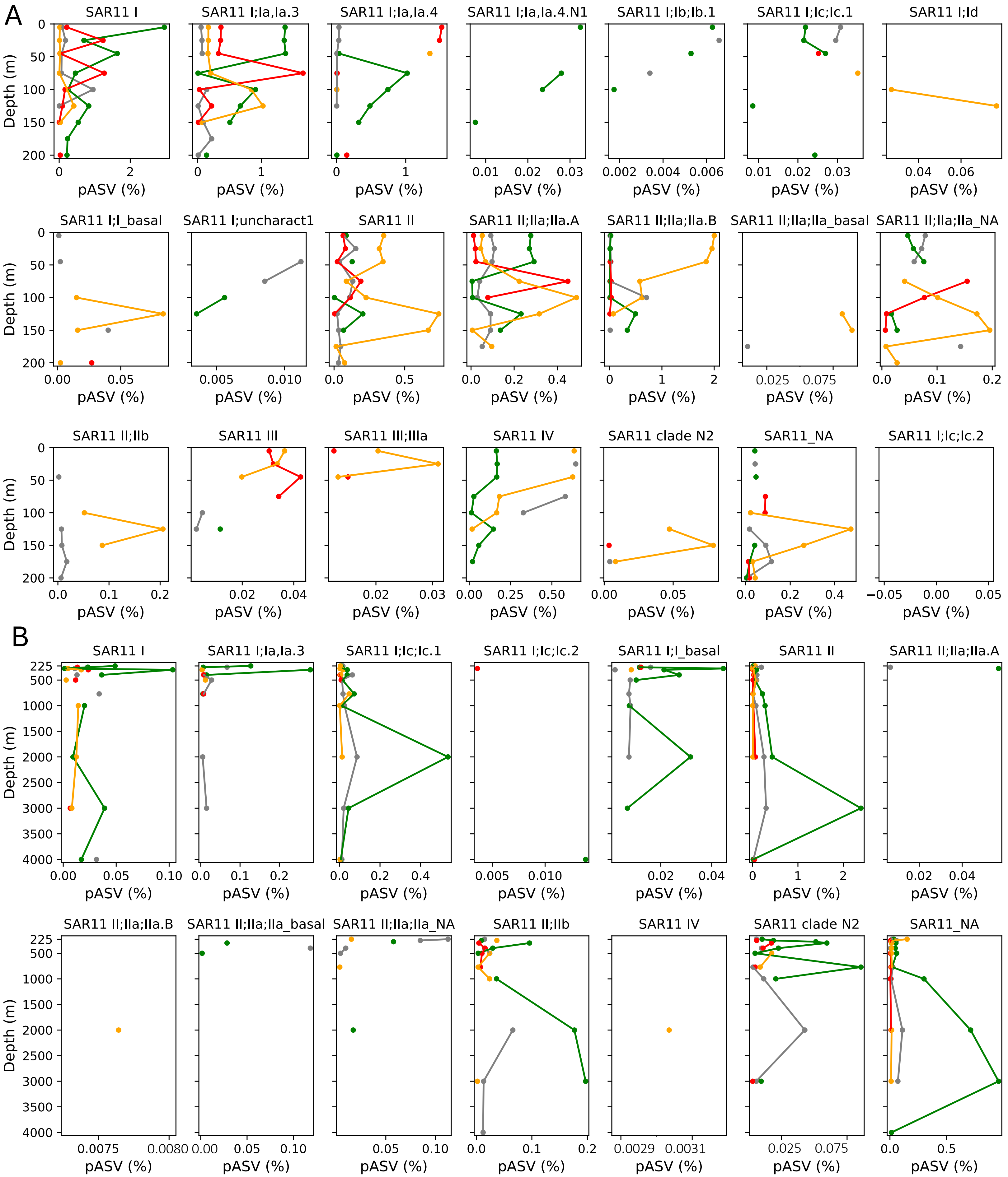

Supplementary Figure 9. Depth profiles of time-averaged percentages of SAR11 pASV seasonal ecotypes, having winter (grey color), spring (green color), summer (red color) or fall (orange color) abundance maxima in the euphotic zone A) or the dark ocean B).

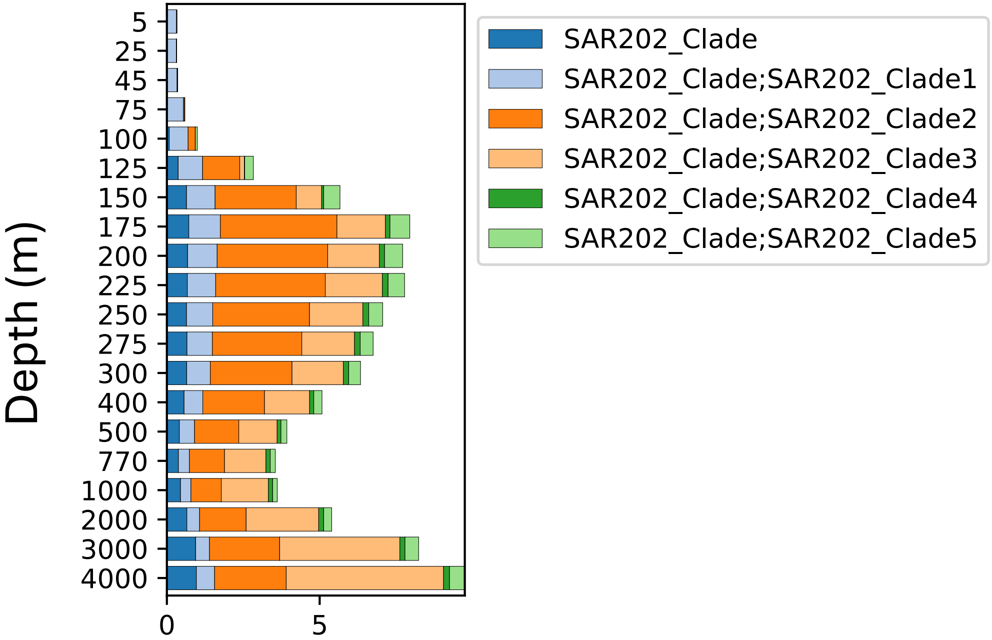

Supplementary Figure 10. Depth ASV patterns of SAR202 ecotypes detected in the water column.

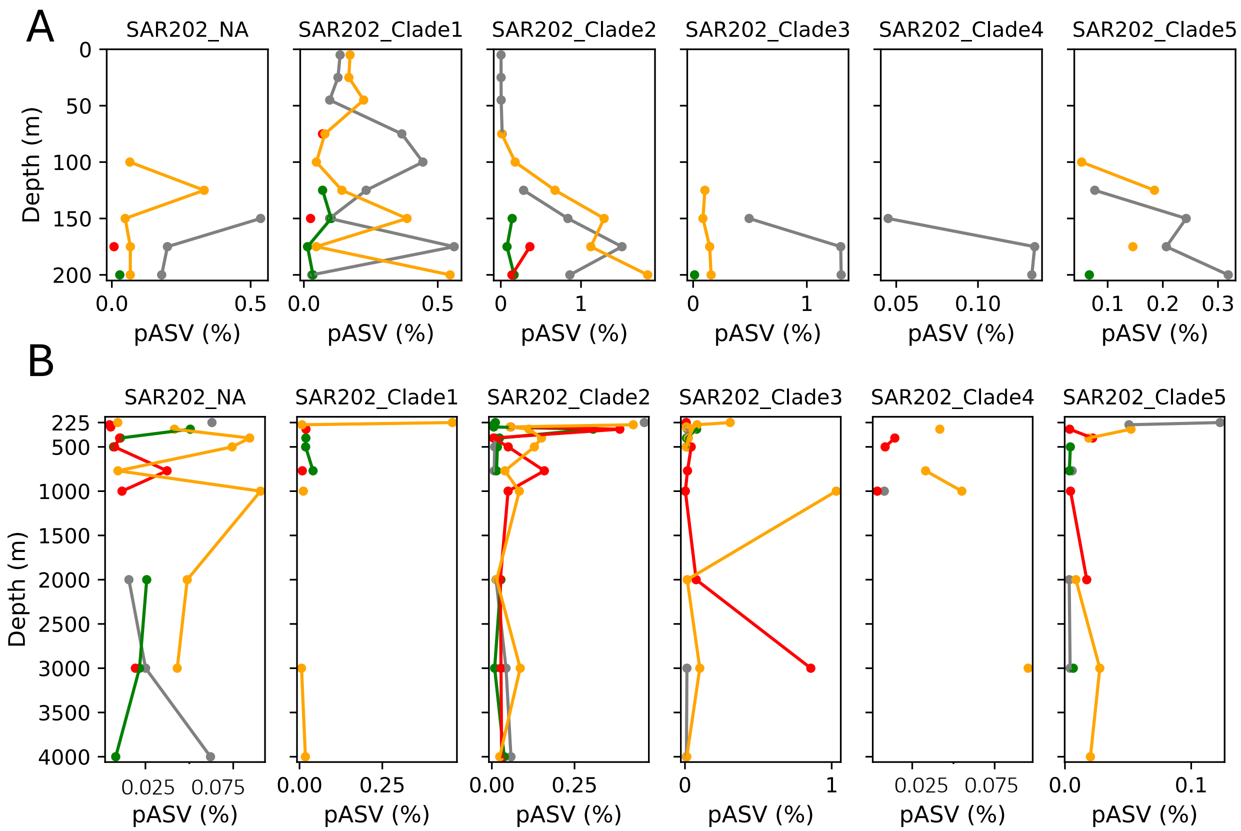

Supplementary Figure 11. Depth profiles of time-averaged percentages of SAR202 pASV ecotypes having winter (grey color), spring (green color), summer (red color) or fall (orange color) seasonal abundance maxima in the euphotic zone A) or the dark ocean B).

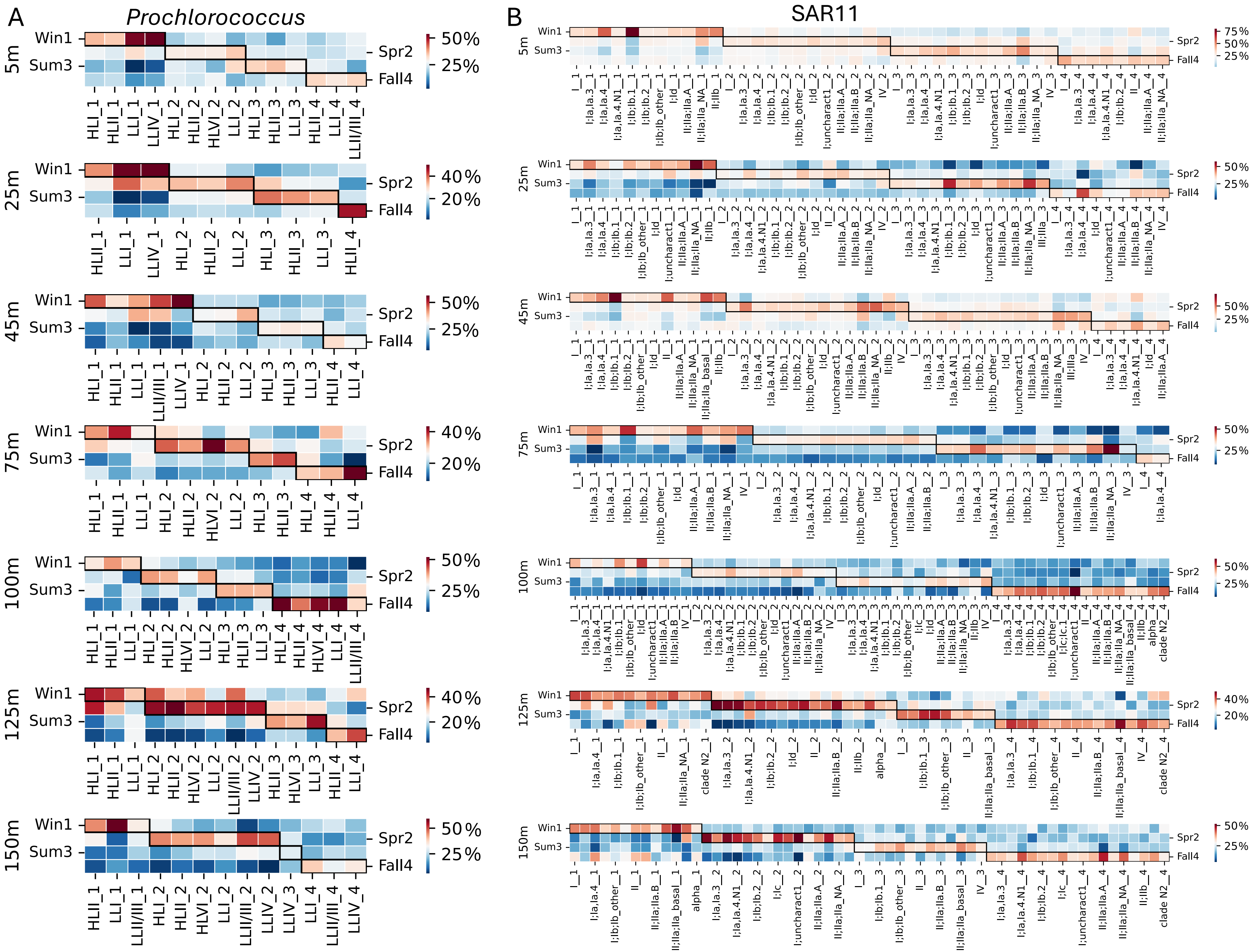

Supplementary Figure 12. Heatmap of COG0012 mOTU season-averaged percentages of winter (_1 on x-axis)-, spring (_2 on x-axis)-, summer (_3 on x-axis)- and fall (_4 on x-axis)-peaking seasonal ecotypes in *Prochlorococcus* A) and SAR11 B) subclades that show annual periodicity in the euphotic zone. Win1, Spr2, Sum3 and Fall4 on y-axis mean winter-, spring-, summer- and fall-averaged percentages of seasonal ecotype. Color bar was shown in each plot.

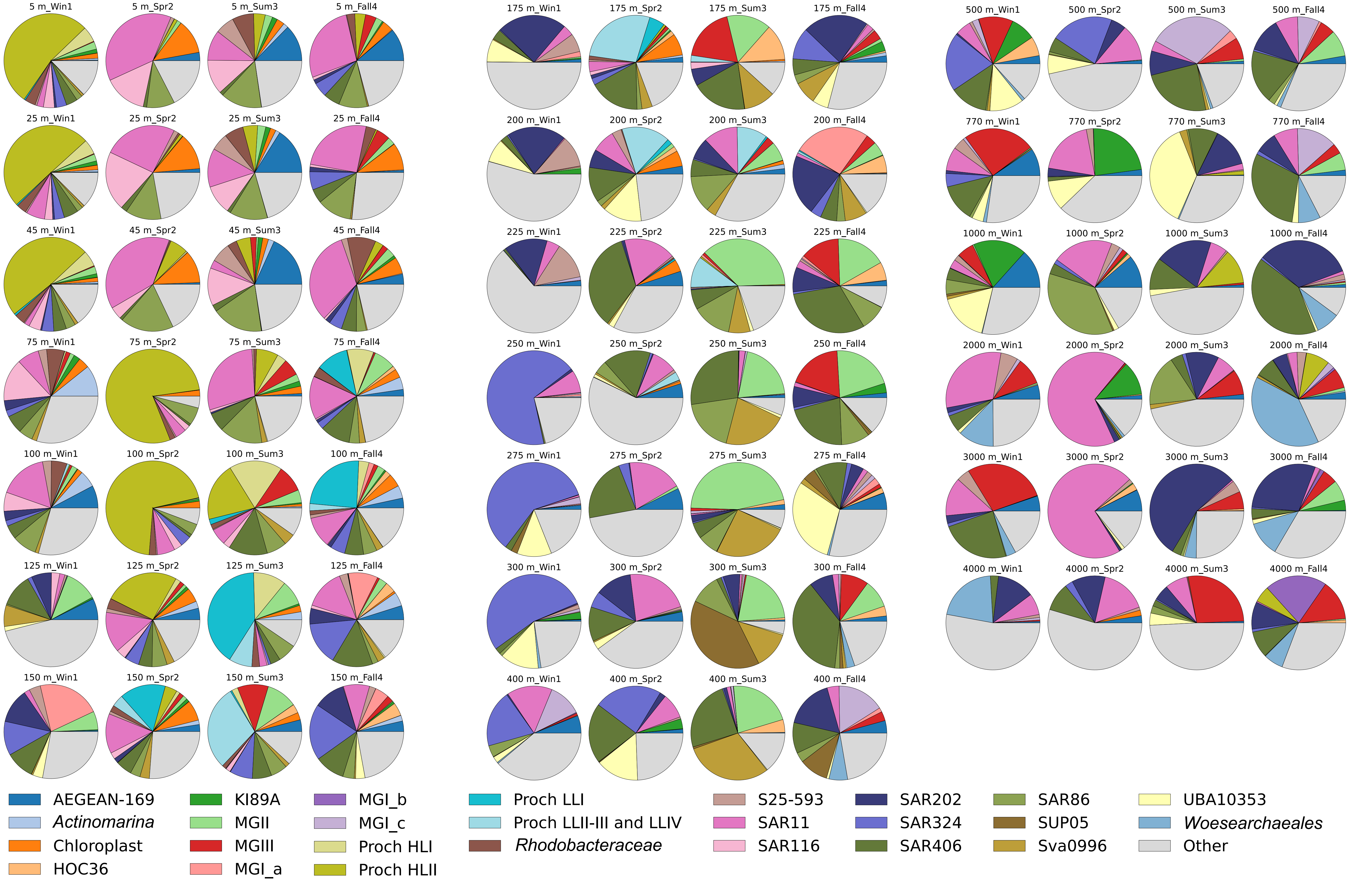

Supplementary Figure 13. The pASV Taxonomic patterns of winter (Win1)-, spring (Spr2)-, summer (Sum3) -, and fall (Fall4)-peaking seasonal ecotypes of dominant planktonic prokaryotes having annual periodicity at the given water column depths shown. MGI_a: *Nitrosopelagicus*; MGI_b: *Nitrosopumilus*; MGI_c: unknown *Nitrosopumilaceae*.

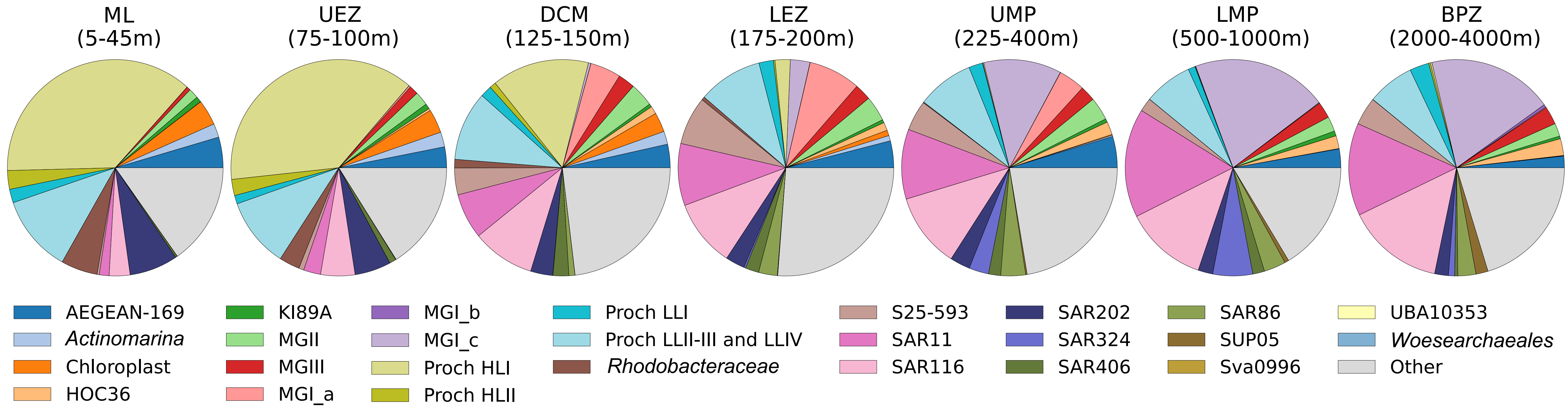

Supplementary Figure 14. The ASV taxonomic patterns of dominant planktonic prokaryotes detected in each depth region. MGI_a: *Nitrosopelagicus*; MGI_b: *Nitrosopumilus*; MGI_c: unknown *Nitrosopumilaceae*. ML: Mixed Layer; UEZ: Upper Euphotic Zone; DCM: Deep Chlorophyll Maximum; LEZ: Lower Euphotic Zone; UPM: Upper Mesopelagic Zone; LMP: Lower Mesopelagic Zone; BPZ: Bathypelagic Zone.

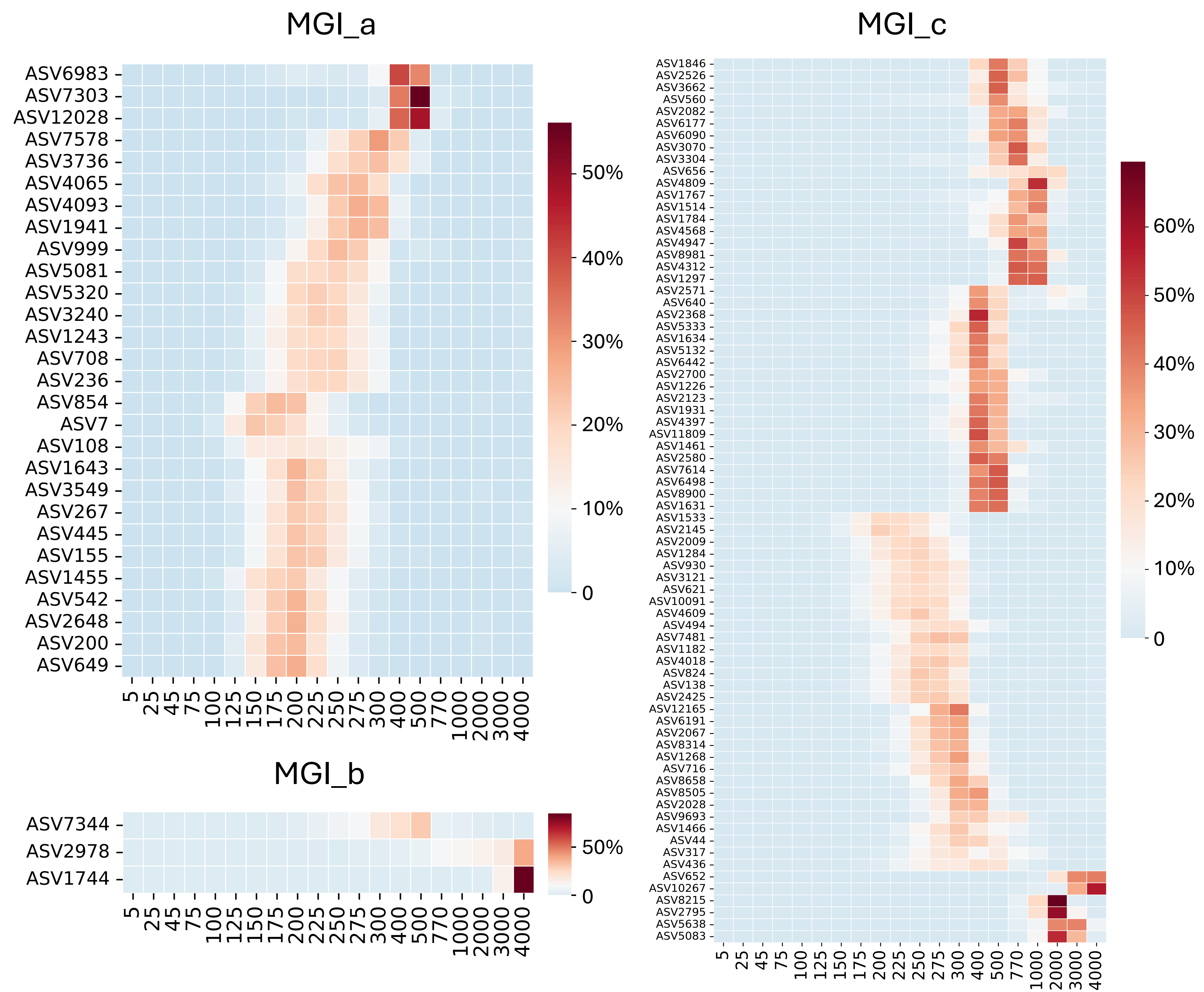

Supplementary Figure 15. Depth pattern of all MGI pASVs, relative to the total abundance of each pASV throughout the water column. MGI_a: *Nitrosopelagicus*; MGI_b: *Nitrosopumilus*; MGI_c: unknown *Nitrosopumilaceae*.

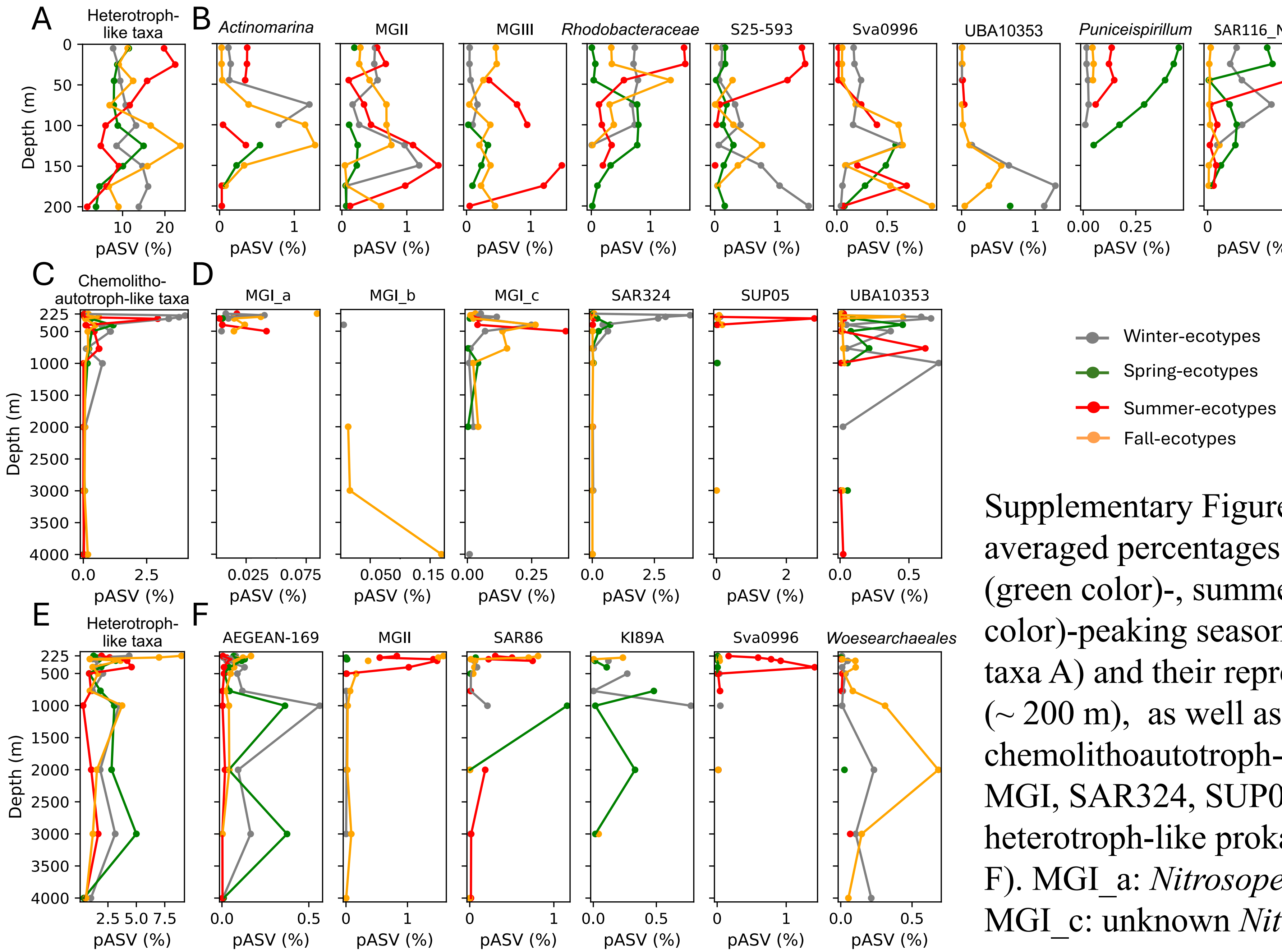

Supplementary Figure 16. Depth pASV profiles of time-averaged percentages of winter (grey color)-, spring (green color)-, summer (red color)- and fall (orange color)-peaking seasonal ecotypes in heterotroph-like taxa A) and their representatives B) in the euphotic zone (~ 200 m), as well as dark ocean (200 m ~) chemolithoautotroph-like prokaryotes C) including MGI, SAR324, SUP05 and UBA10353 D), and heterotroph-like prokaryotes E) and their representatives F). MGI_a: *Nitrosopelagicus*; MGI_b: *Nitrosopumilus*; MGI_c: unknown *Nitrosopumilaceae*.

Supplementary Table 1. Samples mislabeled in 3 out of 74 cruises are corrected.

Original Sample Name	Corrected Sample Name
275-100	275-175
275-175	275-100
332-200	332-100
332-225	332-125
332-250	332-150
332-275	332-175
332-100	332-200
332-125	332-225
332-150	332-250
332-175	332-275
332-1000	332-300
332-2000	332-400
332-3000	332-500
332-4000	332-1000
332-300	332-2000
332-400	332-3000
332-500	332-4000
342-200	342-4000
342-175	342-3000
342-150	342-2000
342-125	342-1000
342-100	342-770
342-75	342-500
342-45	342-400
342-25	342-300
342-3000	342-275
342-4000	342-250
342-5	342-225
342-2000	342-200
342-1000	342-175
342-770	342-150
342-500	342-100
342-400	342-75
342-300	342-45
342-250	342-25
342-225	342-5

Supplementary Table 2. Periodic analysis of Shannon and Richness indices using RAIN. The bold is statistically significant with RAIN $p \leq 0.05$. The season that the diversity peaks in is only provided for the bold ones.

	Shannon		Richness	
	RAIN p value	Diversity peak season	RAIN p value	Diversity peak season
5	0.23	NA[a]	**3.82E-09**	Winter
25	0.44	NA[a]	**2.41E-06**	Winter
45	0.09	NA[a]	**9.94E-09**	Winter
75	**3.65E-03**	Fall	0.91	NA[a]
100	**2.76E-06**	Fall	0.24	NA[a]
125	**1.97E-03**	Fall	0.58	NA[a]
150	0.87	NA[a]	**4.58E-05**	Summer
175	0.91	NA[a]	**2.60E-03**	Summer
200	**8.13E-03**	Spring	**1.04E-04**	Spring
225	0.36	NA[a]	0.10	NA[a]
250	**8.93E-03**	Spring	**1.51E-07**	Spring
275	0.40	NA[a]	0.90	NA[a]
300	**0.02**	Spring	**6.88E-04**	Spring
400	**0.01**	Spring	**2.93E-07**	Spring
500	0.83	NA[a]	**0.01**	Summer
770	0.88	NA[a]	0.86	NA[a]
1000	0.55	NA[a]	0.19	NA[a]
2000	0.84	NA[a]	0.38	NA[a]
3000	0.22	NA[a]	0.25	NA[a]
4000	0.11	NA[a]	**4.14E-03**	Spring

[a]The annual periodicity of diversity is not significant with Rain $p > 0.05$.
