# Supplementary material for: Seasonality drives temporal niche partitioning of pelagic prokaryotes
Source: ISME J. 2026 Apr 9;20(1):wrag062. doi: 10.1093/ismejo/wrag062 (PMC13082231; doi:10.1093/ismejo/wrag062)
Supplement: wrag062_Supplemental_Files [file Suppl12_new.pdf › Suppl12_new.pdf]

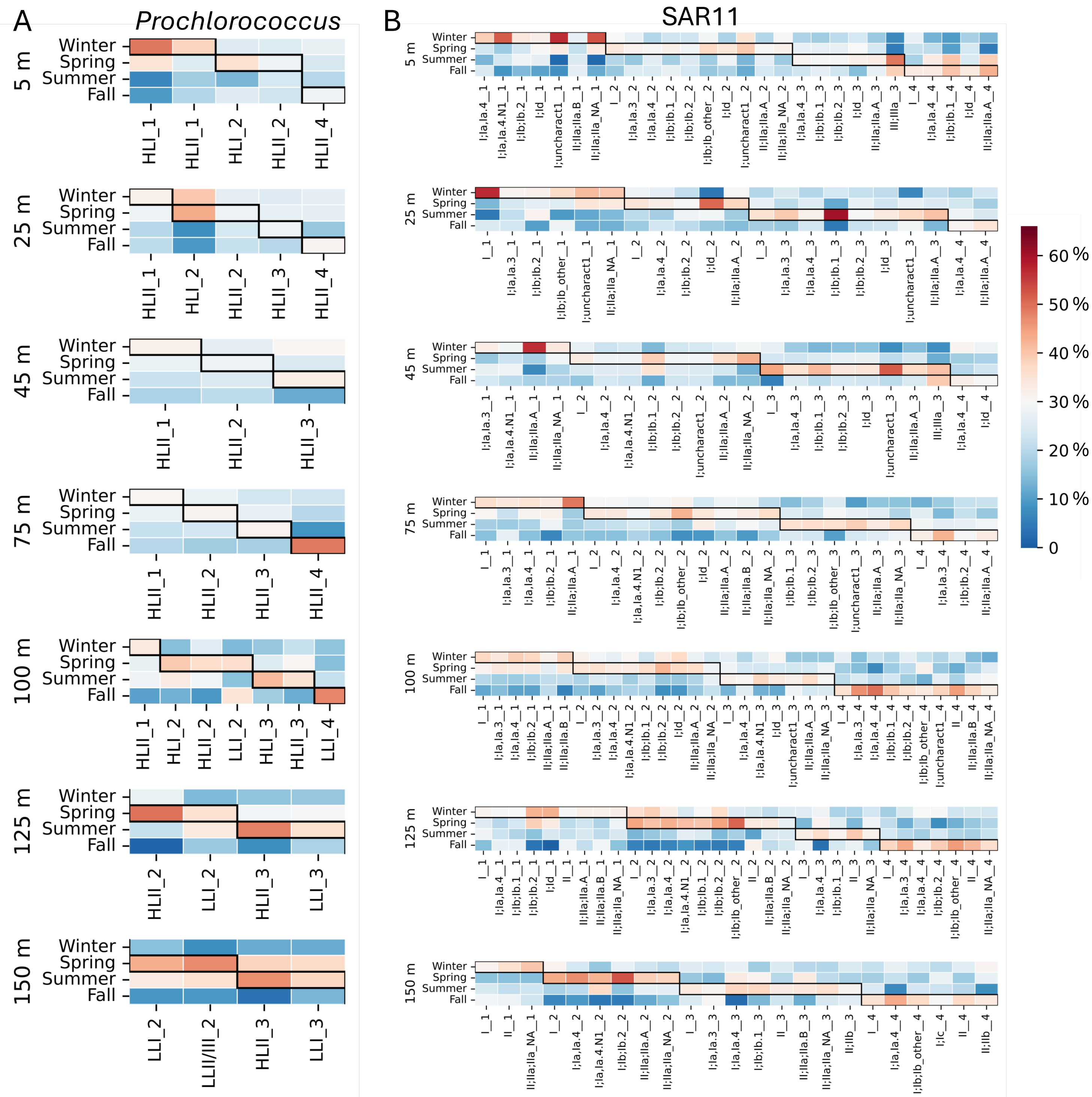

Supplementary Figure 12. Heatmap of COG0012 mOTU season-averaged percentages of winter (_1 on x-axis)-, spring (_2 on x-axis)-, summer (_3 on x-axis)- and fall (_4 on x-axis)-peaking seasonal ecotypes in *Prochlorococcus* A) and SAR11 B) subclades that show annual periodicity in the euphotic zone. Win1, Spr2, Sum3 and Fall4 on y-axis mean winter-, spring-, summer- and fall-averaged percentages of seasonal ecotype. Color bar was shown in each plot.
